# O-GlcNAcylation of UBAP2L regulates stress granule formation and sunitinib resistance in clear cell renal cell carcinoma

**DOI:** 10.1186/s13046-025-03534-0

**Published:** 2025-09-30

**Authors:** Jiajun Xing, Baochao Li, Songbo Wang, Zengjun Wang, Chenkui Miao

**Affiliations:** Department of Urology, The First Affiliated Hospital of Nanjing Medical University, No 300 Guangzhou Road, Nanjing, China

**Keywords:** UBAP2L, Renal cell carcinoma, Patient-derived xenograft, Sunitinib resistance, O-GlcNAcylation, Stress granule formation

## Abstract

**Background:**

Sunitinib resistance is one of the main reasons for the poor prognosis of clear renal cell carcinoma (ccRCC). Moreover, Stress granules (SGs) was found to enhance the stress adaptation capability of tumor cells, becoming an important mechanism for drug resistance in various cancers.

**Methods:**

We developed sunitinib-resistant patient-derived xenograft (PDX) and organoid (PDO) models to investigate sunitinib resistance in ccRCC. Proteomic analysis identified UBAP2L as a key mediator of this resistance. To explore its role in stress granule formation and sunitinib resistance, we conducted both in vitro and in vivo studies. We further elucidated the regulatory mechanisms of UBAP2L O-GlcNAcylation using immunoprecipitation, mass spectrometry, modification-based proteomics, RNA sequencing (RNA-seq), and RNA immunoprecipitation sequencing (RIP-seq).

**Results:**

In this study, enrichment of UBAP2L was elucidated to be significantly associated with sunitinib-resistant ccRCC patient-derived xenograft (PDX) model. Functional experiments showed that UBAP2L protected ccRCC from apoptosis and promoted ccRCC prolifecation and angiogenesis upon sunitinib treatment, thus enhancing drug resistance of ccRCC cells. Furthermore, mechanistic investigation demonstrated that O-GlcNAcylation of UBAP2L promoted its protein stability via inhibiting TRIM37-mediated ubiquitination and it regulated stress granule formation, thereby enhancing the mRNA stability of Melk and activating the PI3K signaling pathways.

**Conclusions:**

These results validated the significant roles of O-GlcNAcylation of UBAP2L in ccRCC sunitinib resistance, which provided an innovative theoretical basis for the clinical diagnosis and therapy of ccRCC.

**Supplementary Information:**

The online version contains supplementary material available at 10.1186/s13046-025-03534-0.

## Graphical abstract

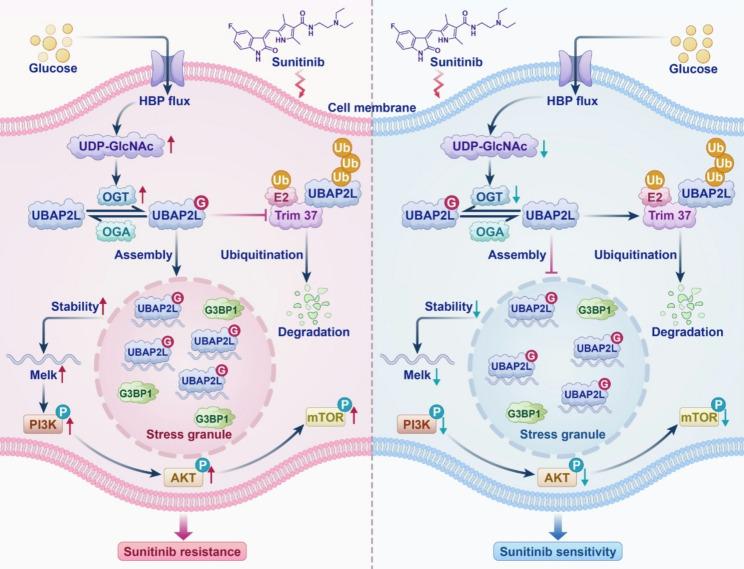

## Introduction

Renal cell carcinoma (RCC), a heterogeneous malignancy originating from the renal epithelium, accounts for approximately 3% of adult cancers globally, with rising incidence rates observed over the past decade [1].

Clear cell RCC (ccRCC), the predominant histological subtype, is characterized by the inactivation of the von Hippel-Lindau (VHL) tumor suppressor gene and subsequent activation of hypoxia-inducible factors (HIFs), which drive angiogenesis and tumor progression [2]. While surgical resection remains curative for localized disease, approximately 30% of patients subsequently develop metastatic recurrence, necessitating systemic therapies [3]. Sunitinib, a multi-targeted tyrosine kinase inhibitor (TKI) that targets vascular endothelial growth factor receptors (VEGFRs) and platelet-derived growth factor receptors (PDGFRs), has become the cornerstone of first-line treatment for advanced ccRCC [4, 5]. Despite initial therapeutic efficacy, the majority of patients develop innate or acquired resistance within 6–15 months, leading to disease progression and poor survival outcomes [6]. This phenomenon underscores the urgent need to elucidate the molecular mechanisms underlying resistance and to develop predictive biomarkers or combinatorial strategies.

Emerging evidence suggests that sunitinib resistance arises from a multifaceted interplay of oncogenic signaling adaptations and remodeling of the tumor microenvironment (TME) [7]. Key mechanisms include the compensatory activation of alternative pro-survival pathways (MET/AXL and mTOR signaling), metabolic reprogramming toward aerobic glycolysis, and epigenetic dysregulation of drug efflux transporters [8–11]. For instance, sustained overexpression of glucose-6-phosphate dehydrogenase (G6PD) in resistant tumors enhances nucleotide synthesis, while CD44-mediated epithelial-mesenchymal transition (EMT) fosters invasive phenotypes [12, 13]. Furthermore, TME components such as hypoxia, immune cell infiltration, and extracellular matrix stiffness contribute to drug evasion by altering vascular permeability and immune surveillance [14–17]. However, despite these advances, a comprehensive framework integrating genomic, metabolic, and microenvironmental factors remains elusive.

Current therapeutic strategies aim to overcome resistance through combination therapies targeting co-activated pathways. Preclinical models demonstrate the synergistic effects of sunitinib with mTOR inhibitors (temsirolimus) or epigenetic modulators, while ongoing clinical trials are investigating immune checkpoint inhibitors in refractory ccRCC [18–20]. Nevertheless, the absence of validated biomarkers for patient stratification or treatment response prediction limits clinical translation.

Stress granules (SGs) represent pivotal adaptive mechanisms in eukaryotic cells under environmental stress, dynamically regulating mRNA translation and degradation to enhance survival [21]. Emerging evidence underscores their critical role in tumor biology, particularly in modulating therapeutic resistance via stress-adaptive pathways. Among the molecular components governing SG dynamics, ubiquitin-associated protein 2-like (UBAP2L) has emerged as a central regulator. Structural analyses reveal that UBAP2L forms distinct SG cores through its ubiquitin-associated (UBA) and Arg-Gly-Gly (RGG) motifs, functioning both collaboratively and independently of the canonical nucleator G3BP1 [22–24]. This functional duality enables UBAP2L to initiate SG assembly upstream of G3BP1/2, as evidenced by its capacity to nucleate SG-like condensates even under stress-native conditions.

Clinically, UBAP2L overexpression correlates with advanced tumor phenotypes and poor prognosis in multiple carcinomas [25, 26]. Mechanistically, it promotes cancer proliferation through SG-mediated mRNA sequestration and translation reprogramming. This functional nexus suggests that UBAP2L-driven SG formation may serve as a survival scaffold during chemotherapeutic stress. Particularly in hepatocellular carcinoma, UBAP2L overexpression enhances malignant phenotypes, while PRMT1-mediated methylation establishes a dynamic equilibrium between pro-survival SG assembly and the oncogenic functions of methylated UBAP2L [21, 27]. Such duality implies context-dependent roles in therapy resistance, where stress-induced SG formation may transiently protect tumor cells from cytotoxic agents, while constitutive UBAP2L activity drives proliferative signaling. Understanding this balance requires further exploration of how SG dynamics mediated by UBAP2L’s post-translational modifications influence drug responses across various cancer subtypes.

O-GlcNAcylation, a nutrient-sensitive post-translational modification, dynamically regulates protein functions by covalently attaching to serine/threonine residues through the enzymatic cycling of O-GlcNAc transferase (OGT) and O-GlcNAcase (OGA) [28, 29]. As a metabolic integrator linked to glucose and nucleotide metabolism via the hexosamine biosynthetic pathway, this modification modulates transcriptional regulation, signal transduction, and cell cycle progression by interacting competitively or cooperatively with phosphorylation. Emerging evidence highlights its pivotal role in pathological conditions, particularly cancer progression. Notably, hyper-O-GlcNAcylation of TOP2A at Ser1469 disrupts cell cycle checkpoints by suppressing p27 expression, facilitating S-phase progression in Adriamycin-resistant breast cancer cells [30]. Such dysregulation impairs chemotherapeutic efficacy through altered protein interactions with cell cycle regulators, revealing a mechanistic nexus between metabolic adaptation and drug resistance. Consequently, targeting O-GlcNAc-mediated signaling cascades represents a promising strategy for overcoming therapeutic resistance in malignancies.

Herein, we constructed the acquired sunitinib-resistant ccRCC PDX model and cell models and demonstrated that UBAP2L regulates sunitinib resistance in renal cell carcinoma by modulating stress granule assembly. Mechanistically, UBAP2L undergoes O-GlcNAcylation mediated by OGT, enhancing its protein stability by suppressing TRIM37-triggered ubiquitination. As a critical assembly factor of stress granules, O-GlcNAcylated UBAP2L enhanced the mRNA stability of Melk and activating the PI3K signaling pathways through its dynamics, thereby promoting therapeutic resistance. Our findings suggest that targeting the O-GlcNAcylation of UBAP2L represents a novel therapeutic strategy for treating clear cell renal cell carcinoma.

## Method

### Clinical samples and database

We obtained transcriptional and clinical data from the official website of The Cancer Genome Atlas (TCGA). All patients diagnosed with ccRCC were recruited from the Department of Urology at the First Affiliated Hospital of Nanjing Medical University at the time of surgery. The collection and analysis of tumor samples were conducted in accordance with the approval of the Ethics Committee of Nanjing Medical University, and all patients provided informed consent. The histological features of the tissue were independently examined by two pathologists according to WHO standards.

### Sunitinib-resistant RCC cell models and PDX models

Cells (786-O and Caki-1) were intermittently exposed to increasing sunitinib concentrations (starting at 5µM) during logarithmic growth phases. Drug concentration was incrementally escalated to 20µM following confirmed tolerance. Resistance (786-O-R and Caki-1-R) were confirmed via CCK-8 assays under sunitinib pressure.

Fresh tumor tissues from treatment-naive ccRCC patients were obtained. Tumor specimens (3–5 mm³ pieces) were washed in cold PBS containing 10% fetal bovine serum (FBS) and penicillin/streptomycin and subcutaneously implanted into 6-week-old male NOD-scid-IL2rg⁻/⁻ (NSG) mice. First-generation (P0) PDXs were passaged to subsequent generations (P1-P3) by re-implanting tumor fragments into new cohorts of mice.

To induce sunitinib resistance, mice bearing established PDXs (tumor volume: ∼200 mm³) were orally administered sunitinib (40 mg/kg/day) for 6 weeks. Resistance was confirmed by tumor regrowth after an initial response phase (≥ 90% volume reduction).

### RCC Patient-derived organoid (PDO) construction

Cut renal cell cancer tissue samples into approximately 1–3 mm³ tissue blocks under sterile conditions. Add digestion solution and digest at 37 °C for 15–30 min, then terminate digestion. Filter using a 100 μm filter. Collect the filtrate into a 15 ml centrifuge tube, and after a 5-minute enrichment centrifugation at 300 g and 4 °C, remove the supernatant. Add matrix gel to the cell pellet, mix by pipetting, and then perform plating. Place the prepared culture plates in a 37 °C incubator for 40–60 min to gel, then add 500–750 µl organoid culture medium for culture.

### Cell lines and culture

The human renal cell carcinoma cell lines 786-O (CVCL_1051) and Caki-1 (CVCL_0234) were purchased from Procell Life Science & Technology (Wuhan, China). The human embryonic kidney cell line 293T (CVCL_0063) were purchased from American Type Culture Collection (ATCC, USA). The 786-O (Procell) and Caki-1 (Procell) were cultured in RPMI-1640 medium (Gibco) supplemented with 10% fetal bovine serum (FBS, Gibco), 100 U/mL penicillin, and 100 µg/mL streptomycin. The 293T (ATCC) was maintained in Dulbecco’s Modified Eagle Medium (DMEM, Gibco) containing 10% FBS. All cell lines were authenticated by short tandem repeat (STR) profiling and routinely tested for mycoplasma contamination using MycoAlert kits every 6 months. Cells were incubated at 37 °C in a humidified atmosphere with 5% CO_2_. For 786-O-R and Caki-1-R, parental cells were chronically exposed to sunitinib (Selleck) in RPMI-1640, alternating between drug-supplemented and drug-free media, as previously described.

### Stress granule fractionation and enrichment

In brief, 786-O and Caki-1 cell lines were treated with 2µM sunitinib for 24 h. The cells were subjected to a brief treatment with digitonin to render the membrane permeable, followed by homogenization and lysis using a specific lysis buffer. In a low-temperature environment, impurities and unpelleted components were removed by gradually increasing the centrifugation speed and duration (8500 g for 2 min, followed by 18000 g for 20 min), ultimately yielding a high-purity stress granule (SG) core component (S850) [31].

### Co-Immunoprecipitation, silver staining, and MS analysis

Cells were lysed in ice-cold RIPA buffer (Thermo Fisher Scientific) containing 1% Triton X − 100, 150 mM NaCl, 50 mM Tris - HCl (pH 7.5), and protease inhibitor cocktail. Lysates were centrifuged at 12,000 × g for 15 min at 4 °C to remove debris. Supernatants were pre - cleared with Protein A/G - agarose beads (Selleck) and incubated with specific primary antibodies or IgG control overnight at 4 °C with rotation. Immunocomplexes were captured with Protein A/G magnetic beads (Thermo Fisher Scientific) at 4 °C for 2 h. Beads were washed 5 times with lysis buffer, resuspended in SDS sample buffer, and boiled at 95 °C for 10 min. Proteins were separated by SDS - PAGE and analyzed by immunoblotting with appropriate secondary antibodies. Non - specific IgG controls were included for antibody specificity confirmation.

For Co-Immunoprecipitation (Co-IP) to identify interacting proteins, precipitated protein complexes were visualized by silver staining. Protein samples from Co-IP assays were separated by 10% SDS-PAGE. After electrophoresis, gels were fixed and processed using a Fast Silver Stain Kit (Beyotime, P0017S) as per the manufacturer’s instructions. Silver-stained bands were visualized under white light. Selected bands of interest were excised for subsequent mass spectrometry (MS) identification. Parallel negative controls with non-specific IgG antibodies were set for specificity confirmation. MS analysis was performed by Shanghai Applied Protein Technology.

### Antibodies, chemicals, plasmids and lentivirus

The antibodies used as follows: β-Actin (66009-1-Ig, Proteintech), UBAP2L (A300-533 A, BETHYL, ), Melk (2274, Cell Signaling Technology), GAPDH (2118, Cell Signaling Technology), Flag (14793, Cell Signaling Technology), cleaved caspase-3 (TA327916, Origene), caspase-3 (19677-1-AP, Proteintech), Ubiquitin (20326, Cell Signaling Technology), O-GlcNAc (9875, Cell Signaling Technology), HA (3724, Cell Signaling Technology), Myc (2276, Cell Signaling Technology).

The chemicals used as follows: Sunitinib (S7781), 3-MA (S2726), MG132 (S2619), Cycloheximide (S7418) were purchased from Selleck. The sources of the remaining reagents are marked clearly in each section.

Plasmids and Lentivirus were constructed by GENECHEM Biotech and Corues Biotechnology. The sequences of all short-hairpin RNAs (shRNAs) and sgRNA are listed in Supplementary Table S1.

### RNA Immunoprecipitation

RNA Immunoprecipitation (RIP) was performed using the Magna RIP™ Kit (17–700, Millipore). The 10^7^ cells were harvested and lysed in RIP lysis buffer containing protease inhibitors. The lysates were incubated with Protein A/G magnetic beads pre-coated with 5 µg of UBAP2L antibody (BETHYL, A300-533 A) or control IgG (Millipore) at 4 °C for 6 h under gentle rotation. After immunoprecipitation, the bead-antibody complexes were washed six times with RIP wash buffer. To digest bound proteins, the complexes were treated with Proteinase K buffer at 55 °C for 30 min. RNA was then extracted from the supernatant and quantified by qRT-PCR. Input RNA (10% of total lysate) served as a normalization control. All primer sequences are listed in Supplementary Table S2.

### Glutathione S-transferase (GST) pull-down assay

Glutathione S-transferase (GST) pull-down assay was performed to examine protein-protein interactions. GST or GST-tagged recombinant proteins were expressed in Escherichia coli BL21 and immobilized onto Anti-GST Magnetic Beads (Beyotime, P2138) in binding buffer. After incubation with cell lysates overnight at 4 °C with gentle rotation, beads were washed 5 times with ice-cold lysis buffer. Bound proteins were eluted using SDS loading buffer and analyzed by SDS-PAGE followed by Coomassie blue staining and immunoblotting with specific antibodies. Control experiments with GST alone were conducted in parallel to confirm interaction specificity.

### Subcutaneous xenograft tumor model

Male NOD-SCID mice (4 weeks old) were subcutaneously injected with 1 × 10^7^ tumor cells resuspended in 100 µL Matrigel mixture. Mice were humanely sacrificed when tumors reached a maximum volume of 1,500 mm³ or at the experimental endpoint. Subcutaneous tumors were excised, photographed, and weighed for further analysis.

### Apoptosis assay and detection of caspase-3 activity and TUNEL staining assay

For apoptosis analysis, cells were harvested, washed with PBS, and stained with Annexin V-APC and 7-AAD using apoptosis kits (KeyGEN BioTECH, KGA1106) according to the manufacturer’s instructions. After 15 min incubation in the dark, samples were analyzed by flow cytometry. Caspase3 activity was measured via spectrophotometry using a kit (Absin, abs50025), where enzymatic cleavage of the caspase-3-specific substrate (pNA) was quantified by absorbance at 405 nm. For TUNEL staining, apoptotic cells were labeled using a TUNEL assay kit (Vazyme).

### Human umbilical vein endothelial cell (HUVEC) tubule formation assay

HUVECs were co-cultured with treated cells. 48-well plates were precoated with 150 µL of Matrigel (Corning, USA) per well and polymerized at 37 °C for 30 min. Subsequently, HUVECs (1 × 10^4^cells/well) resuspended in 200 µL conditioned medium were seeded onto the polymerized Matrigel and incubated for 3 h at 37 °C. Tube structures in randomly selected fields were imaged using an inverted microscope (Leica), and tubule length and branch points were quantified by ImageJ software (version 1.53).

### CCK-8 assay and colony formation assay

Cells were seeded into 96-well plates at a density of 2 × 10^3^ cells per well and cultured under specific experimental conditions. At indicated time points (24, 48, 72, or 96 h), the medium was replaced with 100µL of CCK-8 working solution (Apexbio) and incubated for 1.5 h at 37 °C. Absorbance was measured at 450 nm using a microplate reader. For drug sensitivity analysis, cells were treated with gradient concentrations of compounds (0-128µM sunitinib) for 48 h. Cell viability was quantified via CCK-8 assay as described above. A dose-response curve was generated to calculate the half-maximal inhibitory concentration (IC50) using nonlinear regression analysis.

Cells (1000 cells/well) were seeded into 6-well plates and cultured for 7–14 days. Colonies were fixed with 4% paraformaldehyde and stained with 0.5% crystal violet (Beyotime) for 30 min. Visible colonies (> 50 cells per colony) were counted using ImageJ software.

### Dual-luciferase reporter assay

Cells were seeded in 6-well plates and transfected at 70–80% confluence with the indicated dual-luciferase reporter plasmids (Corues Biotechnology), using transfection reagents such as Lipo3000 (Thermo Fisher Scientific). After 48 h of incubation, cells were lysed, and luciferase activities were measured using a Dual-Luciferase Reporter Assay System (Vazyme) according to manufacturer protocols. Firefly luciferase activity was normalized to Renilla luciferase activity to account for variations in transfection efficiency.

### Statistical analysis

Consistent with established literature, experimental data are presented as mean ± standard deviation, with the number of experimental samples detailed in this paper. Statistical analyses were conducted using GraphPad 8.0. Data from two groups were assessed using a two-tailed t-test, while significant differences among more than two groups were determined using one-way ANOVA. *P*-values < 0.05, < 0.01, and < 0.001 were denoted by*, ** and ***, respectively. A lack of significant difference was indicated as ns.

## Result

### Overexpression of UBAP2L is associated with acquired resistance to sunitinib in ccRCC

**Fig. 1:**
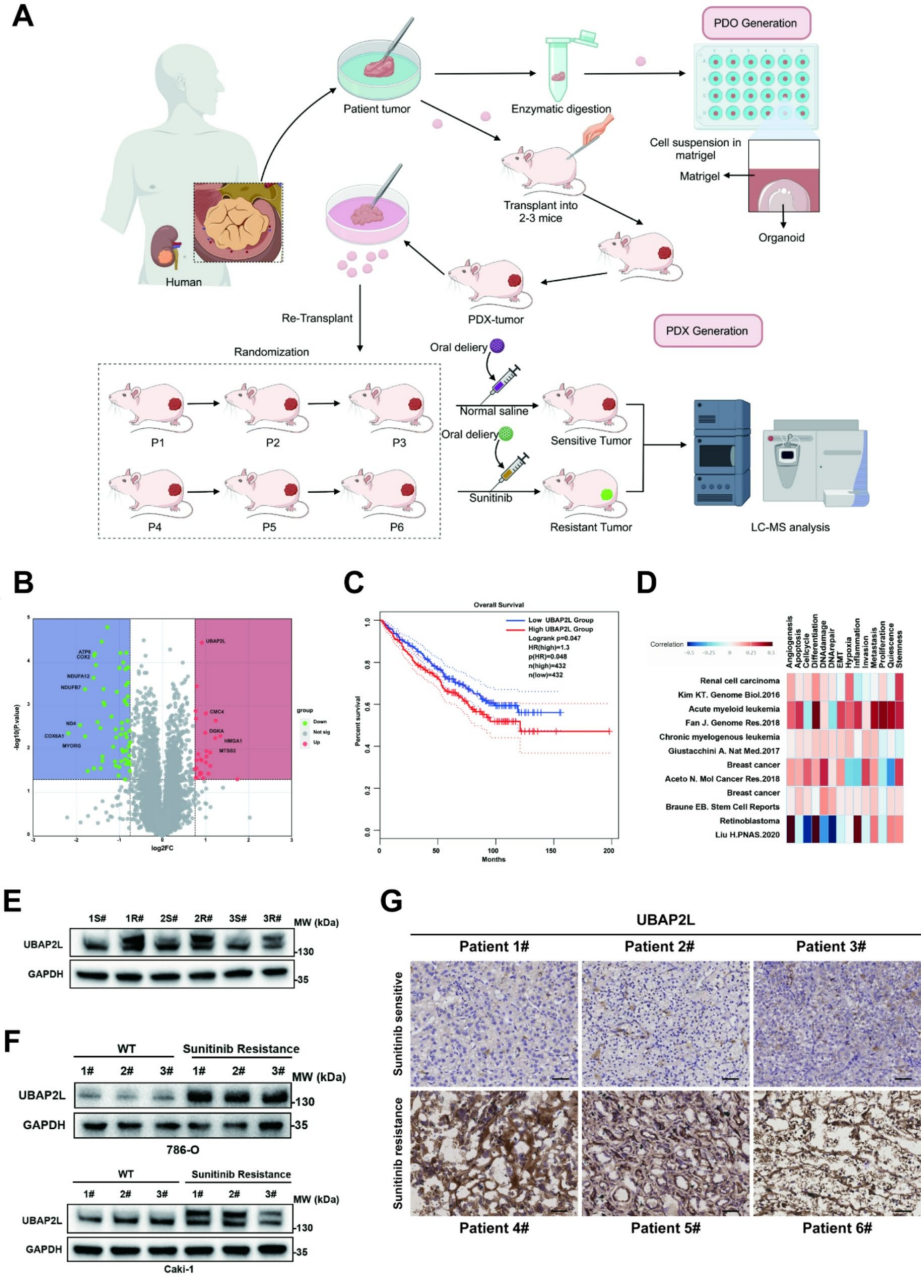
UBAP2L is highly expressed in sunitinib-resistant ccRCC PDX and is associated with poor prognosis. (**A**) Schematic formulation of ccRCC patient-derived xenograft (PDX) and Patient-derived organoid (PDO) model establishment and therapy map of the PDX model. (**B**) Volcano plot of differentially expressed genes identified between sunitinib-resistant and sunitinib-sensitive ccRCC PDX models. Points represent genes, plotted by log_2_fold change (log_2_FC) on the x-axis and *P*-value significance on the y-axis. (**C**) Kaplan-Meier curve for the 2 clusters in the The Cancer Genome Atlas (TCGA) cohort (log-rank *P* = 0.047, HR = 1.3). (**D**) The cancerSEA dataset (http://biocc.hrbmu.edu.cn/CancerSEA/) was used to. analysis the cancer-related function of UBAP2L in various types of malignant tumor. (**E**) The protein level of UBAP2L from ccRCC PDX models with (*n* = 3) or without (*n* = 3) sunitinib resistance was examined by Western blotting analysis. (**F**) The protein level of UBAP2L from 786-O and Caki-1 cells with or without sunitinib resistance (786-O-R and Caki-1-R) was examined by Western blotting analysis. Three replicates were performed. (**G**) Representative images of IHC staining for UBAP2L in two pairs of sunitinib-resistant and sunitinib-sensitive ccRCC tissues. Scale bars, 50 μm

**Fig. 2:**
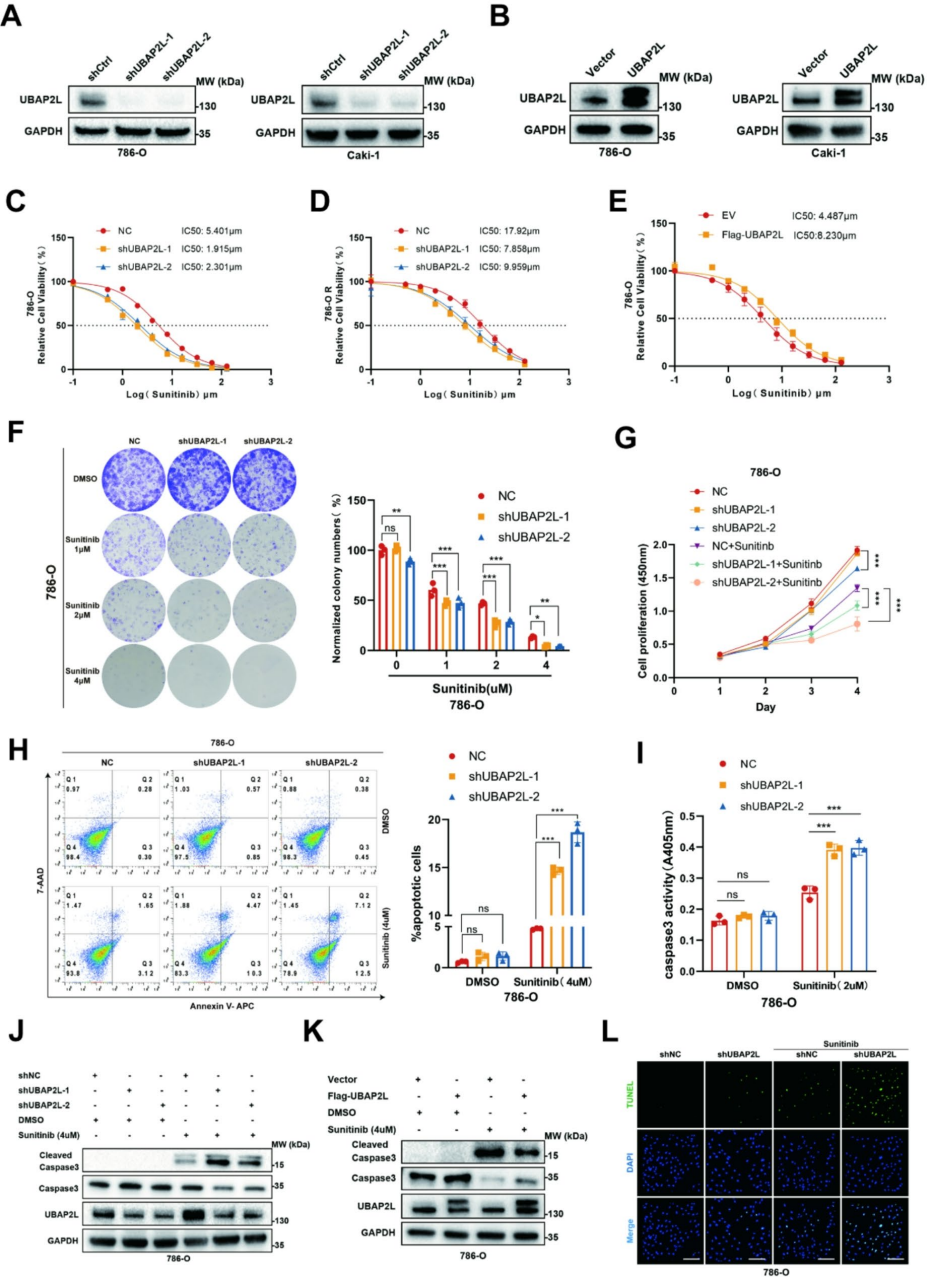
UBAP2L promotes sunitinib resistance of ccRCC cells in vitro. (**A-B**) 786-O and Caki-1 cells were transfected with indicated constructs for 72 h. After puromycin selection, cells were collected for Western blot analysis. (**C-E**) 786-O and 786-O R cells were transfected with indicated constructs for 72 h. After puromycin selection, these cells were treated with a serial dose of sunitinib for 24 h. and subjected to CCK-8 assay. The IC50 values of sunitinib in each group were indicated. (**F**) Colony formation assays upon UBAP2L knockdown 786-O cells after 2-week sunitinib treatment. (**G**) 786-O cells were transfected with indicated constructs for 72 h. After puromycin selection, these cells were treated with or without sunitinib (2µM) for 96 h and subjected to CCK-8 assay. *P* values were determined by two-tailed t test or two-way analysis of variance (ANOVA). *P < 0.05, **P < 0.01, ***P < 0.001. (**H-L**) 786-O cells were transfected with indicated constructs for 72 h. After 24 h puromycin selection, cells were treated with or without sunitinib (2µM or 4µM) for another 24 h. Cells were collected for Flow cytometry analysis of apoptosis (**H**), Caspase 3 activity assay (**I**), Western blot analysis (**J-K**) and TUNEL staining (**L**) Scale bars, 150 μm. Data presents as mean ± SEM with three replicates. Ns, not significant; *P < 0.05, **P < 0.01, ***P < 0.001

To investigate the mechanisms that potentially contribute to the development of sunitinib resistance in ccRCC, we established PDX and patient-derived organoid (PDO) models from samples obtained from a surgically resected ccRCC patient. Six ccRCC PDX models were randomly assigned to two groups (*n* = 3 per group) and treated with either saline or sunitinib (Fig. 1A). While the tumors initially exhibited sensitivity to sunitinib, prolonged treatment led to a loss of therapeutic response by day 75 (Fig. S1A). The growth rate of regrowing tumors in the sunitinib-treated group became comparable to that of untreated controls, indicating the development of drug resistance. This pattern of initial response followed by resistance mirrors the clinical phenotype observed in sunitinib-resistant ccRCC patients. We conducted proteomic sequencing analysis to comprehensively define the changes in protein expression levels associated with the sunitinib-resistant PDX tumor model. The proteomic analysis revealed that a total of 28 proteins were highly expressed in the sunitinib-resistant group (Fig. 1B). To further validate the impact of these 28 highly expressed proteins on sunitinib therapy, we knocked down four main genes: UBAP2L, CMC4, DGKA, and HMGA1. By monitoring cell proliferation rates in the 786-O, 786-O-R, Caki-1, and Caki-1-R cell lines, we found that the knockdown of UBAP2L reduced the sunitinib IC50 and reversed resistance, whereas CMC4, DGKA, and HMGA1 did not exhibit similar effects (Fig. 2C-D, S2A-B and S3D-E). Additionally, to investigate the implications of UBAP2L expression in patient tumors, we analyzed data from TCGA. We found that elevated levels of UBAP2L were associated with significantly poorer survival (hazard ratio (HR) = 1.3, logrank *p* = 0.047) (Fig. 1C). Furthermore, we utilized the CancerSEA dataset (cancer single-cell state atlas) to explore the role of UBAP2L in various types of malignant tumors (Fig. 1D). UBAP2L was positively correlated with hypoxia and angiogenesis, which may explain its association with sunitinib resistance in ccRCC.

To investigate the connection between UBAP2L and acquired sunitinib resistance in ccRCC, we first assessed UBAP2L expression in both drug-resistant and drug-sensitive PDX models. UBAP2L expression levels were significantly higher in the sunitinib-resistant PDX group compared to the control group (Fig. 1E and Fig. S1D). Additionally, we established sunitinib-resistant renal cell carcinoma lines, 786-O-R and Caki-1-R. Compared to their parental cell lines, these resistant cell lines exhibited increased UBAP2L protein expression (Fig. 1F). We examined UBAP2L protein expression by IHC in patients with ccRCC treated with sunitinib. We found that ccRCC patient with high UBAP2L expression showed significant higher resistance to sunitinib as compared with patients with ccRCC with low UBAP2L expression (Fig. 1G). Conversely, no significant difference in UBAP2L mRNA levels was observed among the PDX models (Fig. S1B). Similarly, UBAP2L mRNA levels remained unchanged when comparing drug-resistant cell lines to their parental counterparts (Fig. S1C). Accumulating evidence indicates a close connection between UBAP2L and pharmacotherapy resistance.

### UBAP2L regulates stress granule formation and promotes the resistance to sunitinib of ccRCC in vitro

Initially, we suppressed the expression of UBAP2L using two independent shRNAs targeting UBAP2L (shUBAP2L-1 and shUBAP2L-2) in the 786-O and Caki-1 and their drug-resistant cell lines. Following knockdown, the expression level of UBAP2L in each cell line was assessed through Western blot assays (Fig. 2A and S3A). Additionally, the efficacy of UBAP2L overexpression was validated (Fig. 2B and S3B).

**Fig. 3:**
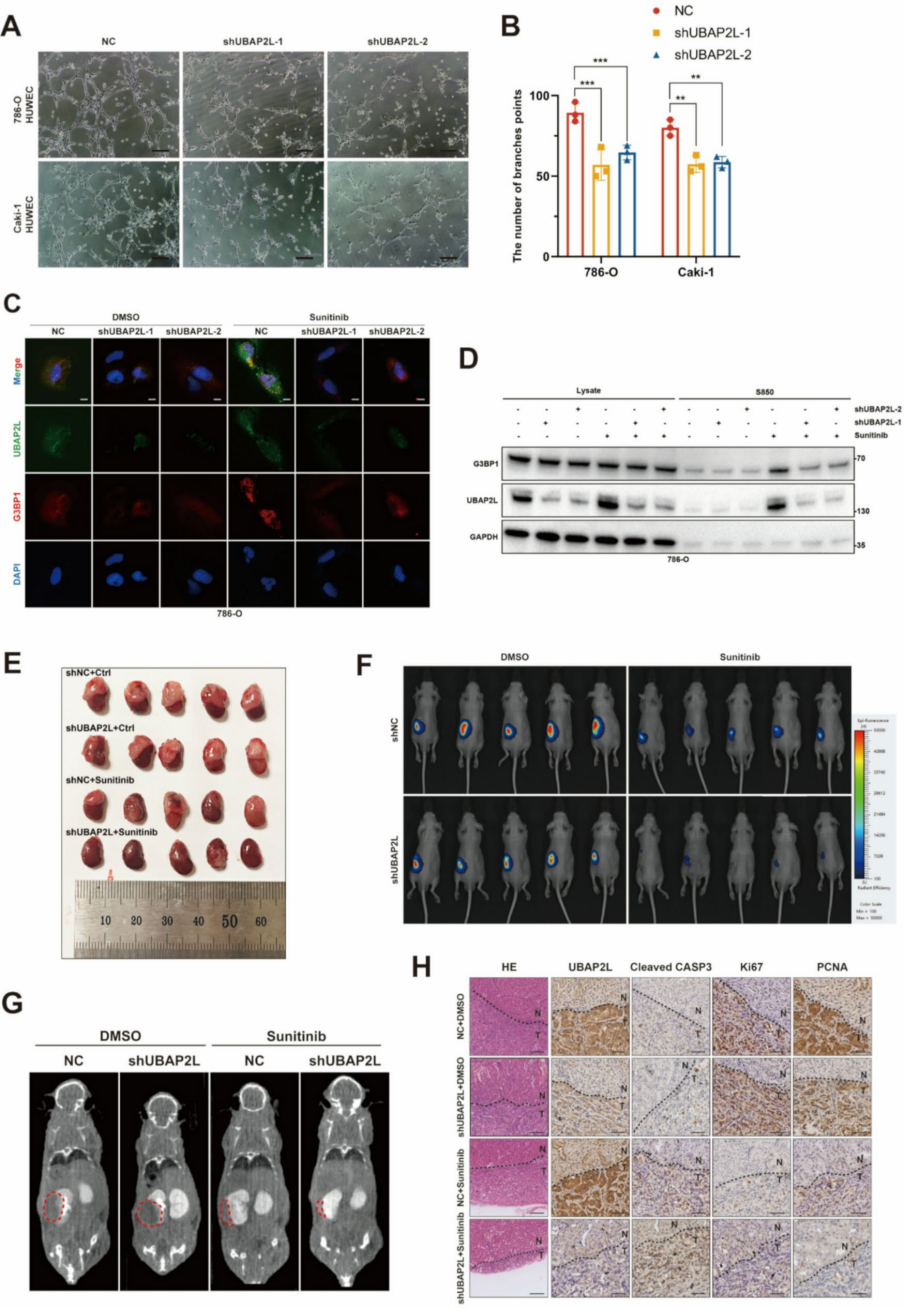
UBAP2L regulates stress granule formation and promoted the resistance to sunitinib of ccRCC in vitro. (**A**) Representative images of tube formation at 8 h after cells were seeded on Matrigel. HUVECs were co-cultured at UBAP2L knockdown cells for 24 h, Scale bar, 25 μm. (**B**) The number of branches points in each group from Fig. 3A were quantified using the Image J software. Data presents as mean ± SEM with three replicates. **P < 0.01, ***P < 0.001. (**C**) 786-O was transfected with control or UBAP2L shRNAs, and 72 h later, and the cells treated with 2µM sunitinib for 24 h were immunostained for UBAP2L together with G3BP1, Scale bars, 10 μm. (**D**) The accumulation of G3BP1 after UBAP2L knockdown and sunitinib treatment in 786-O. SG fractions (S850) were obtained by serial centrifugations, and the samples were analyzed by immunoblotting with the indicated antibodies. Protein levels of G3BP1 and UBAP2Lwere quantified. GAPDH was used as the loading control. (**E**) Representative images of xenograft orthotopic ccRCC model (*n* = 5). (**F**) Representative bioluminescent images of xenograft orthotopic ccRCC model. (**G**) Representative computed tomography images of xenograft orthotopic ccRCC model. (**H**) Representative images of hematoxylin and eosin (**H&E**) and Immunohistochemistry (IHC). Scale bar for H&E, 150 μm. Scale bar for IHC, 50 μm

Subsequently, we examined the sunitinib resistance associated with UBAP2L in vitro using CCK8 assays and colony formation techniques. Notably, UBAP2L knockdown resulted in diminished cell viability, decreased maximal inhibitory concentration (IC50) values, and a reduction in both the number and size of colonies in ccRCC cells following sunitinib treatment (Fig. 2C and F-G, S3D, and S3F-G). In contrast, UBAP2L overexpression elicited an opposing response in ccRCC cells (Fig. 2E and S3C). Furthermore, we observed that the knockdown of UBAP2L significantly increased the sensitivity of 786-O-R and Caki-1-R cells to sunitinib (Fig. 2D, S3E and S3H-I). Additionally, UBAP2L knockdown combined with sunitinib promoted apoptosis (Fig. 2H-L and S4A-F), while UBAP2L overexpression decreased apoptosis and contributed to sunitinib resistance in 786-O and Caki-1 cells (Fig. 2K and S4C). Moreover, the HUVEC tubule formation assay demonstrated that inhibiting UBAP2L expression reduced the angiogenic capability of 786-O and Caki-1 cells (Fig. 3A-B).

Collectively, these findings indicate that UBAP2L is crucial for the proliferation and angiogenesis of ccRCC and plays a pivotal role in regulating the sensitivity of ccRCC cells to sunitinib.

Reports indicated that UBAP2L is a crucial component of stress granules (SGs) and is essential for their assembly [21, 32]. To determine whether UBAP2L localizes to SGs, 786-O and Caki-1 cells treated with 2 µM sunitinib for 24 h were immunostained for UBAP2L along with SG marker proteins, such as G3BP1. As shown in Fig. 3C and Fig. S4H, UBAP2L accumulated into multiple dot-like structures and colocalized with these marker proteins following sunitinib treatment, consistent with previous findings. We subsequently tested whether UBAP2L is required for SG organization. 786-O and Caki-1 cells transfected with UBAP2L shRNA were treated with sunitinib and immunostained for UBAP2L and G3BP1. As shown in Fig. S4G, transfection with UBAP2L shRNAs clearly disrupted SG organization, which corroborates previous reports. Additionally, we conducted SG component enrichment in wild-type and UBAP2L knockdown ccRCC cells, with or without treatment with 2 µM sunitinib for 24 h. Our results indicated that, following sunitinib treatment, UBAP2L knockdown cells exhibited significantly lower levels of G3BP1 accumulation in the SG components compared to wild-type cells. Both shRNAs significantly reduced UBAP2L abundance in wild-type group, while the expression of the SG marker protein G3BP1 remained unaffected (Fig. 3D and S4I). These results demonstrate that UBAP2L is essential for SG formation.

### Targeting UBAP2L synergizes the efficacy of sunitinib in ccRCC treatment

To investigate the effect of UBAP2L and sunitinib in vivo, orthotopic xenograft models in mice were established using 786-O cells. (Fig. 3E). Four weeks after inoculation, sunitinib (40 mg/kg) or DMSO was administered orally once daily for 4 weeks. In vivo tumorigenesis was evaluated in orthotopic xenograft models using bioluminescence imaging (Fig. 3F). An experimental small animal CT scanner was employed to monitor tumor growth in the orthotopic xenograft models (Fig. 3G).

Notably, sunitinib combined with UBAP2L knockdown impressively mitigated tumor growth compared with the control group, whereas UBAP2L knockdown or sunitinib alone exhibited a partial inhibitory effect. The apoptotic marker (cleaved caspase 3) and proliferation marker (Ki67 and PCNA) staining ascertained that UBAP2L knockdown enhance the anti-tumor activity of sunitinib in vivo through the inhibition of proliferation and the promotion of apoptosis (Fig. 3H). Above all, our data suggested that targeting UBAP2L could be used as a potential sensitizer for ccRCC molecular targeting therapy.

### UBAP2L is O-GlcNAcylated by OGT at conserved serine 305

Based on the observed mRNA and protein levels in clinical samples, we found that the increase in UBAP2L protein levels was more pronounced than the increase in mRNA levels in sunitinib-resistant ccRCC cells. This suggests the involvement of post-translational modifications in the regulation of UBAP2L (Fig. 1F and Fig. S1B-C).

**Fig. 4:**
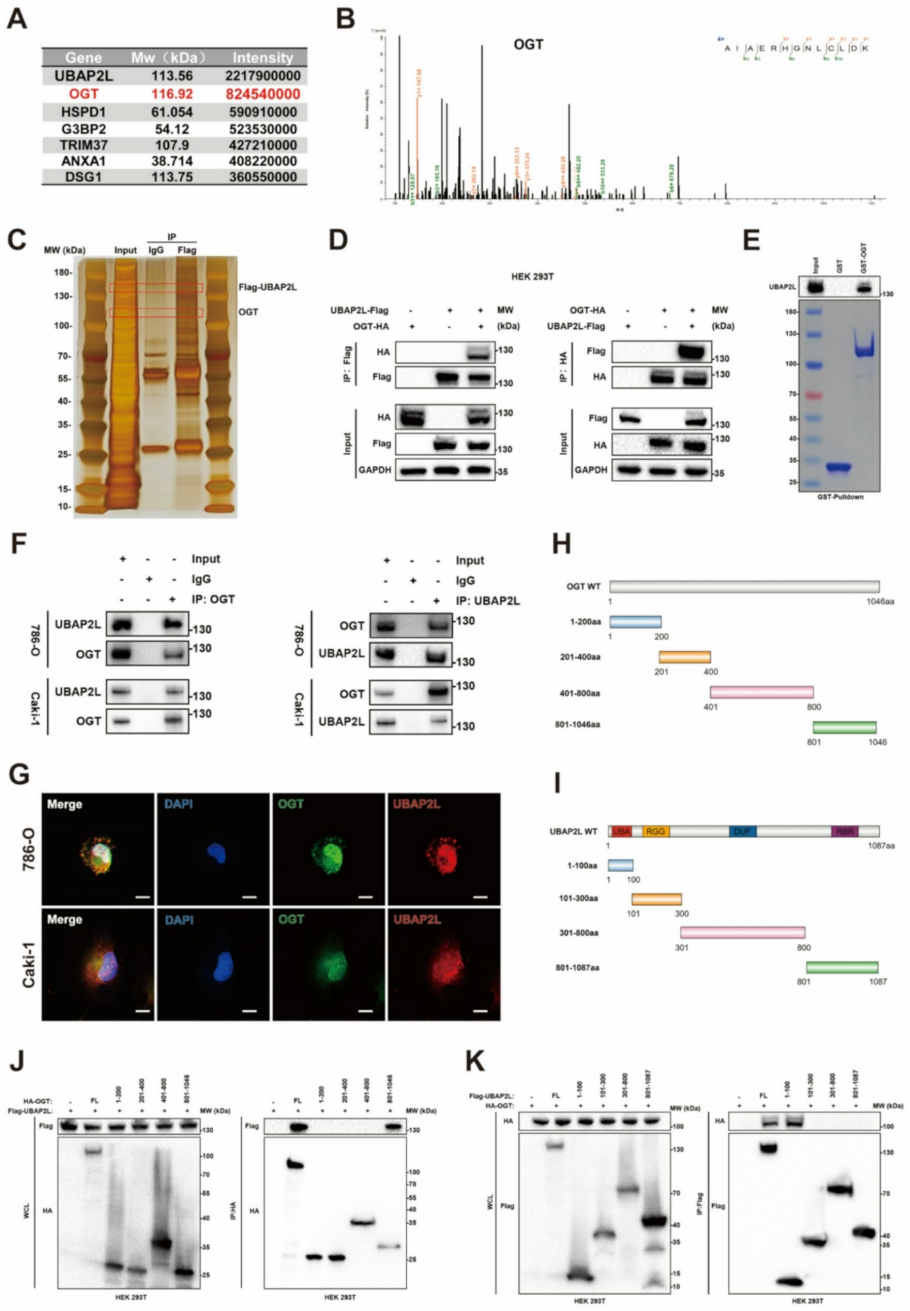
UBAP2L interacts with OGT. (**A**) Mass spectrometry analysis of Flag-UBAP2L-immunoprecipitates in 786-O cell. (**B**) Mass spectrometry analysis of a peptide derived from Flag-UBAP2L-immunoprecipitates to show the potential interaction between UBAP2L and OGT. (**C**) Gel electrophoresis was conducted after using Flag antibody for Flag-UBAP2L-immunoprecipitation, then sliver staining was performed. (**D**) Western blot analysis of ectopically expressed Flag-UBAP2L and HA-OGT reciprocally immunoprecipitated by anti-HA and anti-Flag in 293T cells. (**E**) Western blot analysis of UBAP2L GST-pulldown by GST-OGT recombinant. (**F**) Western blot analysis of endogenous UBAP2L and OGT proteins reciprocally immunoprecipitated by anti-OGT and anti-UBAP2L in 786-O and Caki-1 cells. (**G**) Immunofluorescence confocal microscopy showed the colocalization of UBAP2L and OGT in in 786-O and Caki-1 cells. Scale bar, 10 μm. (**H-I**) Schematic diagram of UBAP2L and OGT and its truncation mutants. (**J**) HEK293T cells were co-transfected with Flag-UBAP2L and HA-tagged FL OGT or its deletion mutants, and cell lysates were analyzed by IP with HA beads followed by IB with antibodies against HA and Flag. (**K**) HEK293T cells were co-transfected with HA-OGT and Flag-tagged FL UBAP2L or its deletion mutants, and cell lysates were analyzed by IP with Flag beads followed by IB with antibodies against HA and Flag

Sliver staining for gel electrophoresis of Flag-UBAP2L-immunoprecipitation in 786-O cells was conducted to show potential interactions based on molecular weight (Fig. 4C). To explore potential regulators of UBAP2L, we identified associated proteins using mass spectrometry following immunoprecipitation (IP) and discovered that OGT interacts with UBAP2L (Fig. 4A-B and S5A). To confirm this interaction, HEK293T cells were transfected or co-transfected with Flag-UBAP2L and HA-OGT, and the proteins were detected with specific antibodies. Our results indicated that UBAP2L and OGT were co-immunoprecipitated (Fig. 4D). Furthermore, we demonstrated that this physical interaction occurs in ccRCC cells as well (Fig. 4F). The GST pull-down assay further validated that UBAP2L binds to immobilized GST-OGT, but not to GST alone (Fig. 4E). Immunofluorescence staining revealed the colocalization of UBAP2L (red) and OGT (green) in the cytoplasm and nucleus of ccRCC cells (Fig. 4G and S5B-C). To elucidate the minimum essential regions required for their interaction, we co-expressed various deletion mutants of UBAP2L and OGT in HEK293T cells (Fig. 4H-I). As shown in Fig. 4J-K, the N-terminal sequence of UBAP2L (amino acids 1-100) and the C-terminal sequence of OGT (amino acids 801–1046) were necessary and sufficient for direct interaction.

**Fig. 5:**
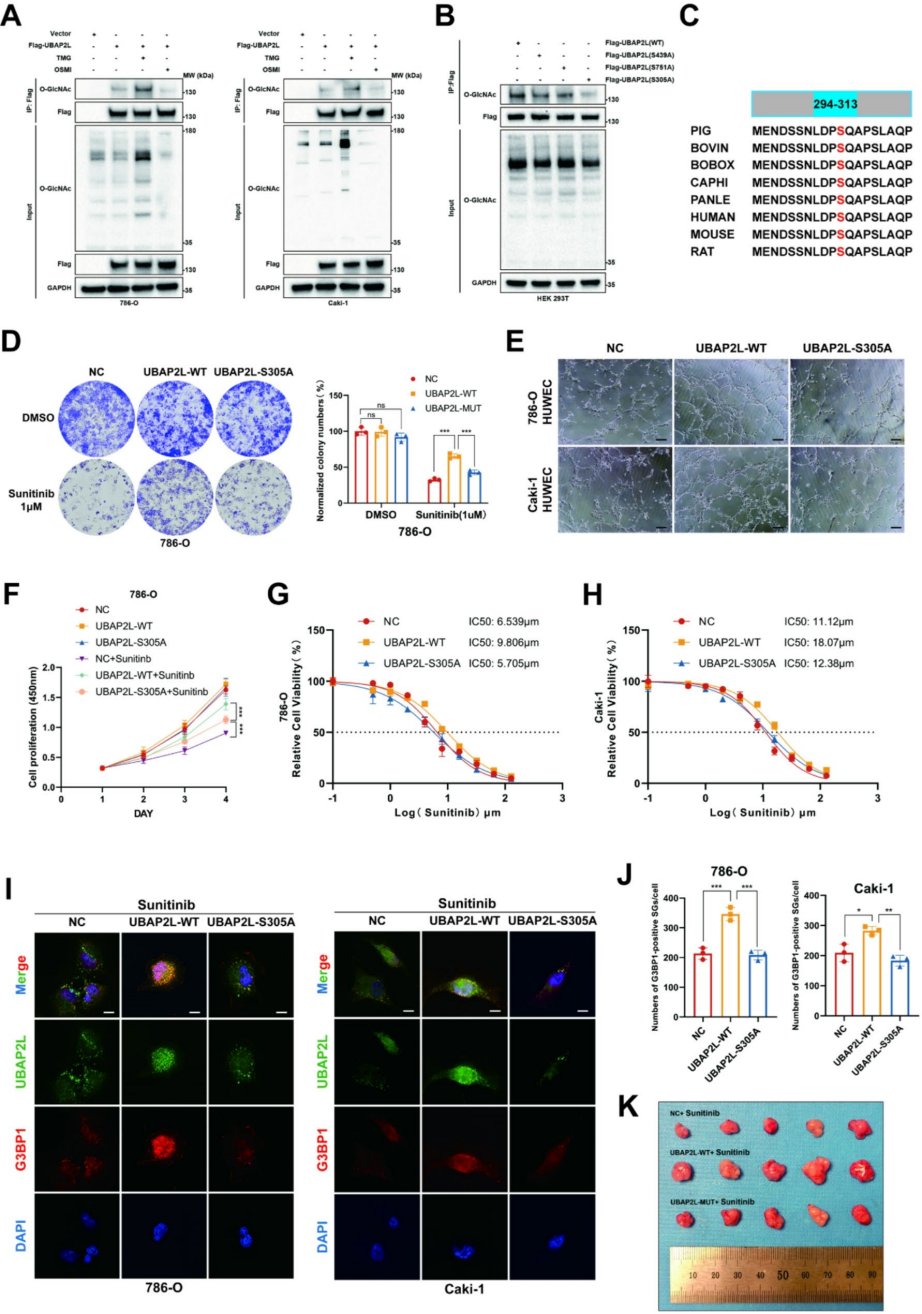
O-GlcNAcylation of UBAP2L at serine 305 regulates stress granule formation and sunitinib resistance in renal cell carcinoma. (**A**) Western blot showing TMG (10µM, 24 h) and OSMI-1 (50µM, 24 h) treatment regulates UBAP2L O-GlcNAcylation level. (**B**) HEK293T cells were transfected with Flag-UBAP2L or its mutants, and cell lysates were analyzed by IP with Flag beads followed by IB with antibodies against O-GlcNAc. (**C**) Alignment of UBAP2L protein sequence among different species. (**D**) 786-O cells infected with Flag-UBAP2L WT/S305A were harvested for colony formation assay after 2-week sunitinib treatment, Data presents as mean ± SEM with three replicates. Ns, not significant, ***P < 0.001. (**E**) Representative images of tube formation at 8 h after cells were seeded on Matrigel. HUVECs were co-cultured at ccRCC cells infected with Flag-UBAP2L WT/S305A for 24 h, Scale bar, 25 μm. (**F**) 786-O cells infected with Flag-UBAP2L WT/S305A were treated with or without sunitinib (2µM) for 96 h and subjected to CCK-8 assay. *P* values were determined by two-tailed t test or two-way ANOVA. ***P < 0.001. (**G-H**) 786-O and Caki-1 cells were transfected with Flag-UBAP2L WT/S305A. After puromycin selection, these cells were treated with a serial dose of sunitinib for 24 h and subjected to CCK-8 assay. The IC50 values of sunitinib in each group were indicated. (**I-J**) 786-O and Caki-1 were transfected with control or Flag-UBAP2L WT/S305A, and 72 h later, and the cells treated with 2µM sunitinib for 24 h were immunostained for UBAP2L together with G3BP1, Scale bars, 10 μm. (**K**) Representative images of xenograft ccRCC model (*n* = 5)

Next, given that OGT is the only enzyme known to catalyze O-GlcNAc modification, we examined whether UBAP2L could be O-GlcNAcylated. Western blot analysis using an anti-O-GlcNAc antibody showed a positive signal in the UBAP2L immunoprecipitated from ccRCC cells (Fig. 5A). Additionally, we manipulated O-GlcNAcylation using TMG and OSMI-1 treatment. Consistent with our hypotheses, increased global O-GlcNAcylation resulted in enhanced UBAP2L O-GlcNAcylation in TMG-treated cells, whereas cells treated with OSMI-1 showed the opposite effect (Fig. 5A). In conclusion, our findings confirmed that UBAP2L can directly bind to and be O-GlcNAcylated by OGT.

Moreover, the O-GlcNAcylated proteins immunoprecipitated from ccRCC cells were analyzed using electron transfer dissociation mass spectrometry, which revealed the S305 site of UBAP2L as the O-GlcNAcylation site. We also employed the YinOYang 1.2 Server to predict other potential O-GlcNAcylation sites on UBAP2L, identifying S439 and S751 as having the highest probability of modification. To ascertain which site is primarily responsible for O-GlcNAcylation, we mutated each site to alanine in HEK293T cells. The most significant reduction in UBAP2L O-GlcNAcylation occurred when the S305 site was mutated (Fig. 5B), suggesting that S305 is the principal O-GlcNAcylation site. Sequence alignment revealed that S305 is highly conserved across different species, reinforcing the notion that it is the main O-GlcNAcylation site (Fig. 5C). Collectively, these results indicate that UBAP2L is O-GlcNAcylated by OGT at the conserved serine residue 305.

In addition, we generated overexpression models of UBAP2L wild type (WT) and a catalytic inactive mutant (MUT) in ccRCC cells. As anticipated, overexpression of UBAP2L-WT increased sunitinib resistance in 786-O and Caki-1 cells compared to both control and UBAP2L-MUT overexpression cells (Fig. 5G-H). Additionally, we assessed the sunitinib-resistant effects of UBAP2L WT and the S305A mutant in vitro via CCK-8 assays, colony formation assays, and tubule formation assays. Significantly, the UBAP2L S305A mutant exhibited reduced cell viability, diminished colony size and number and reduced angiogenic capacity in ccRCC cells (Fig. 5D-F and S5D-F). Subsequently, we constructed an in vivo tumor model to evaluate the effects of the UBAP2L S305A mutant (Fig. 5K). Following sunitinib treatment, the UBAP2L S305A mutant group showed slower tumor growth, smaller tumor size, and lower tumor weight (Fig. S5G-H). Conversely, UBAP2L-WT overexpression markedly enhanced tumor growth and weight after sunitinib treatment.

To investigate whether O-GlcNAcylation of UBAP2L is essential for stress granule (SG) formation, we treated UBAP2L-WT and UBAP2L-S305A overexpression ccRCC cells with sunitinib and performed immunostaining for UBAP2L and G3BP1. As illustrated in Fig. 5I-J and S6A-B, the UBAP2L-S305A mutant clearly disrupted SG formation, consistent with previous findings. Together, these data indicate that OGT-mediated O-GlcNAcylation of UBAP2L at serine 305 regulates stress granule formation and sunitinib resistance in renal cell carcinoma.

### O-GlcNAcylation of UBAP2L promotes its protein stability via inhibiting TRIM37-mediated ubiquitination

**Fig. 6:**
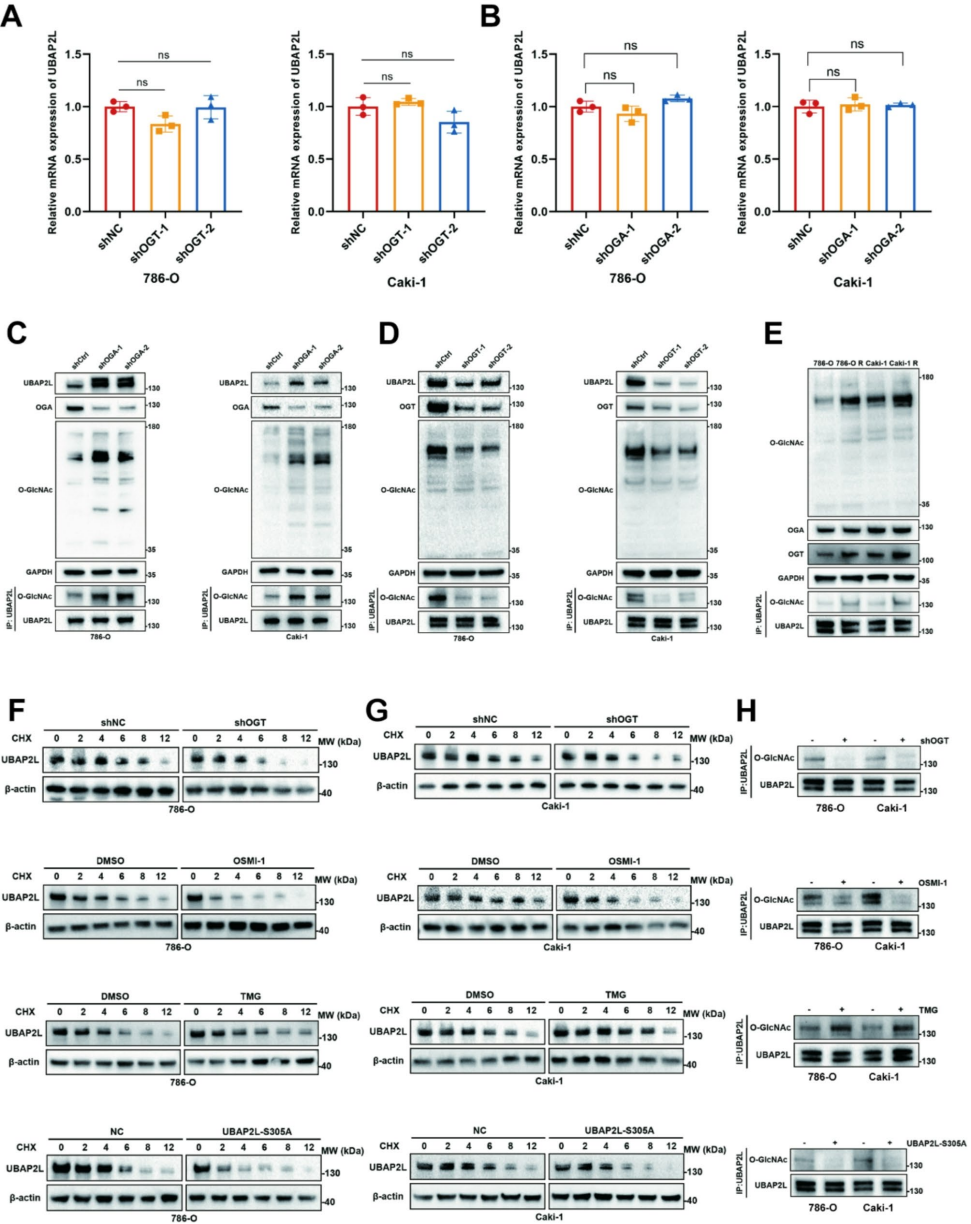
O-GlcNAcylation of UBAP2L promotes its protein stability. (**A-B**) qPCR analysis of UBAP2L expression in shOGT or shOGA or shCtrl ccRCC cells. Data presents as mean ± SEM with three replicates. Ns, not significant. (**C-D**) Western blot analysis of O-GlcNAcylation expression and the level of UBAP2L O-GlcNAcylation in shOGT or shOGA or shCtrl ccRCC cells. (**E**) Western blot analysis of O-GlcNAcylation expression and the level of UBAP2L O-GlcNAcylation in ccRCC cells with or without sunitinib resistance. (**F-G**) The degradation of UBAP2L in ccRCC cells treated with different conditions was evaluated by CHX-chase assay. (**H**) Western blot analysis of UBAP2L expression and O-GlcNAcylation of UBAP2L in ccRCC cells

We subsequently investigated the influence of O-GlcNAcylation on UBAP2L. Our results demonstrated that OGT and the O-GlcNAcylation levels of UBAP2L, were significantly elevated in sunitinib-resistant ccRCC cells (Fig. 6E and S6G). Following the knockdown of OGT in ccRCC cells, we observed a substantial decrease in UBAP2L O-GlcNAcylation, which corresponded with a marked reduction in UBAP2L protein expression (Fig. 6D). Conversely, increased levels of UBAP2L O-GlcNAcylation were associated with heightened UBAP2L protein expression following OGA knockdown (Fig. 6C). However, we noted no significant differences in UBAP2L mRNA levels despite changes in O-GlcNAcylation (Fig. 6A-B). We hypothesized that O-GlcNAcylation affects UBAP2L protein stability and examined UBAP2L expression by modulating its O-GlcNAcylation levels. The expression of the UBAP2L S305 site mutation, OGT knockdown or OSMI-1 resulted in a significant reduction in UBAP2L protein stability compared to controls (Fig. 6F-H). In contrast, treatment of ccRCC cells with TMG led to a significant increase in UBAP2L protein stability (Fig. 6F-H). Overall, these findings indicate that O-GlcNAcylation of UBAP2L promotes its protein stability.

**Fig. 7:**
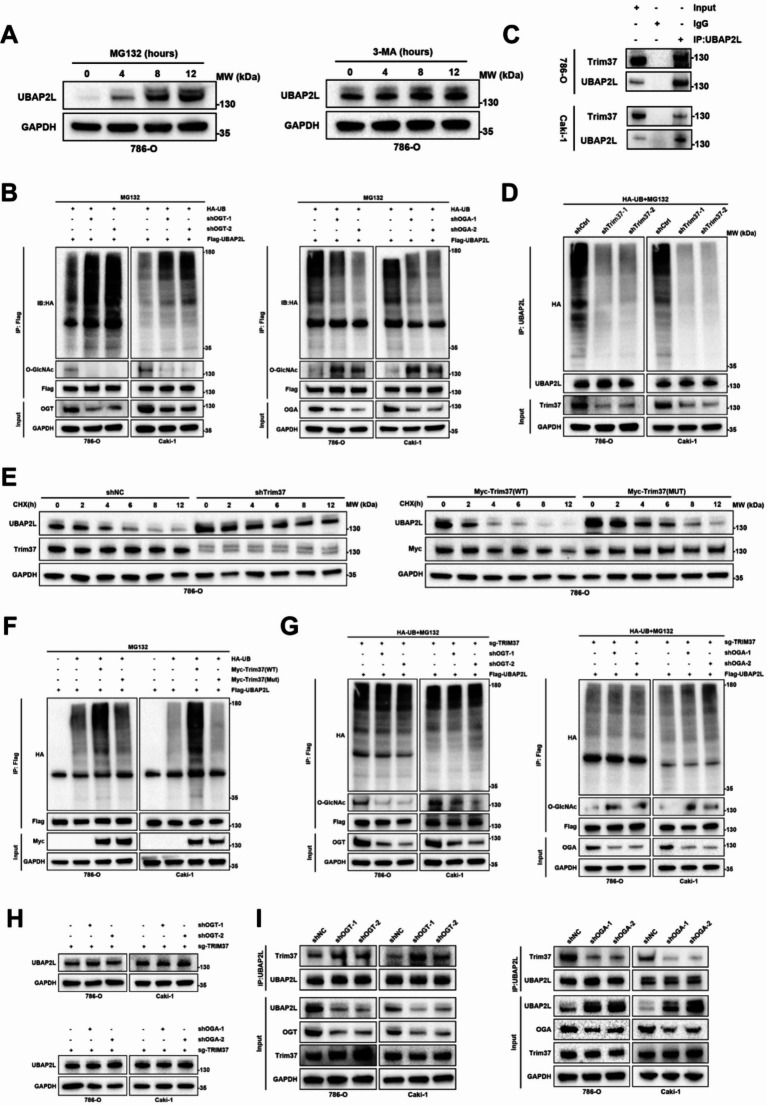
O-GlcNAcylation protects UBAP2L from TRIM37 mediated ubiquitination. (**A**) 786-O cells were treated with or without the proteasome inhibitor MG132 (10 µM,0–12 h) and the autophagy inhibitor 3-MA (10µM, 0–12 h), and then UBAP2L were detected. (**B**) Flag-UBAP2L WT ccRCC cells were transfected with the shCtrl or shOGT or shOGA and HA-Ub, and cell lysates were subjected to IP with Flag antibody, followed by IB with indicated antibodies. Cells treated with 10µM MG132 for 8 h. (**C**) Lysates from ccRCC cells were subjected to IP and immunoblotting analysis with the indicated antibodies. (**D**) ccRCC cells were co-transfected with the shCtrl or shTRIM37 and HA-Ub, and cell lysates were subjected to IP with UBAP2L antibody, followed by IB with indicated antibodies. Cells treated with 10µM MG132 for 8 h. (**E**) 786-O cells transfected with the shCtrl, shTRIM37, TRIM37-WT or TRIM37-MUT were treated with CHX (50 µg/ml), and collected at the indicated times for Western Blot. (**F**) ccRCC cells were co-transfected with Flag-UBAP2L, HA-Ub, and Myc-TRIM37 (WT) or TRIM37 (MUT), and cell lysates were subjected to IP with Flag antibody, followed by IB with indicated antibodies. Cells treated with 10µM MG132 for 8 h. (**G**) Flag-UBAP2L WT ccRCC cells were transfected with the shCtrl or shOGT or shOGA after knockout of TRIM37 and cell lysates were subjected to IP with Flag antibody, followed by IB with indicated antibodies. Cells treated with 10µM MG132 for 8 h. (**H**) ccRCC cells were transfected with the shCtrl or shOGT or shOGA after knockout of TRIM37. UBAP2LL expression was dectected by Western Blot. (**I**) Lysates from ccRCC cells transfected with the shCtrl or shOGT or shOGA were subjected to IP and immunoblotting analysis with the indicated antibodies

Previous studies have shown that UBAP2L is degraded via the ubiquitin-proteasome system (UPS). In ccRCC cells, treatment with the proteasome inhibitor MG132 significantly increased UBAP2L protein levels, while treatment with the autophagy inhibitor 3-MA had no effect on UBAP2L expression, suggesting that UBAP2L degradation occurs primarily through the UPS (Fig. 7A and S6C). Additionally, we found that UBAP2L ubiquitination levels significantly increased following the reduction of O-GlcNAcylation, while elevated O-GlcNAcylation had the opposite effect, suggesting that O-GlcNAcylation of UBAP2L is involved in the regulation of its ubiquitination (Fig. 7B). To identify ubiquitination enzymes that associate with UBAP2L, we conducted an immunoprecipitation assay. Mass spectrometry analysis revealed that TRIM37, a ubiquitin E3 ligase that mediates the ubiquitination of multiple proteins, interacted with UBAP2L (Fig. 4A). We verified the TRIM37-UBAP2L interaction through immunoprecipitation and immunofluorescence assays in ccRCC cells (Fig. 7C and S6D-F). To elucidate TRIM37’s role in UBAP2L regulation, we observed that TRIM37 knockdown increased UBAP2L protein stability, while TRIM37(WT) overexpression, but not the mutants TRIM37(C35/36S), significantly decreased UBAP2L protein stability in ccRCC cells (Fig. 7E and S6H). Given that TRIM37 functions as a ubiquitin E3 ligase, we next assessed its impact on UBAP2L ubiquitination. We found that TRIM37 knockdown significantly reduced UBAP2L ubiquitination in ccRCC cells (Fig. 7D), whereas TRIM37(WT) overexpression increased UBAP2L ubiquitination, in contrast to the mutants TRIM37(C35/36S) which had no effect (Fig. 7F). These findings suggest that TRIM37 is the primary enzyme regulating the ubiquitination-mediated degradation of UBAP2L. Subsequently, we investigated whether O-GlcNAcylation regulates UBAP2L ubiquitination through its influence on TRIM37. We generated TRIM37 knockout ccRCC cells using sgRNA. Neither OGT nor OGA depletion altered UBAP2L expression or ubiquitination levels in TRIM37 knockout ccRCC cells (Fig. 7G-H). Additionally, there were no significant differences in TRIM37 expression levels among ccRCC cells with varying O-GlcNAcylation levels (Fig. 7I). Finally, we examined whether O-GlcNAcylation affected the binding affinity between UBAP2L and TRIM37. Knockdown of OGT enhanced the interaction between TRIM37 and UBAP2L, whereas knockdown of OGA had the opposite effect (Fig. 7I). In conclusion, we established that O-GlcNAcylation of UBAP2L reduces its TRIM37-mediated ubiquitination.

### UBAP2L directly binds Melk and enhances its mRNA stability via regulating stress granule formation to activate PI3K signaling in ccRCC

**Fig. 8:**
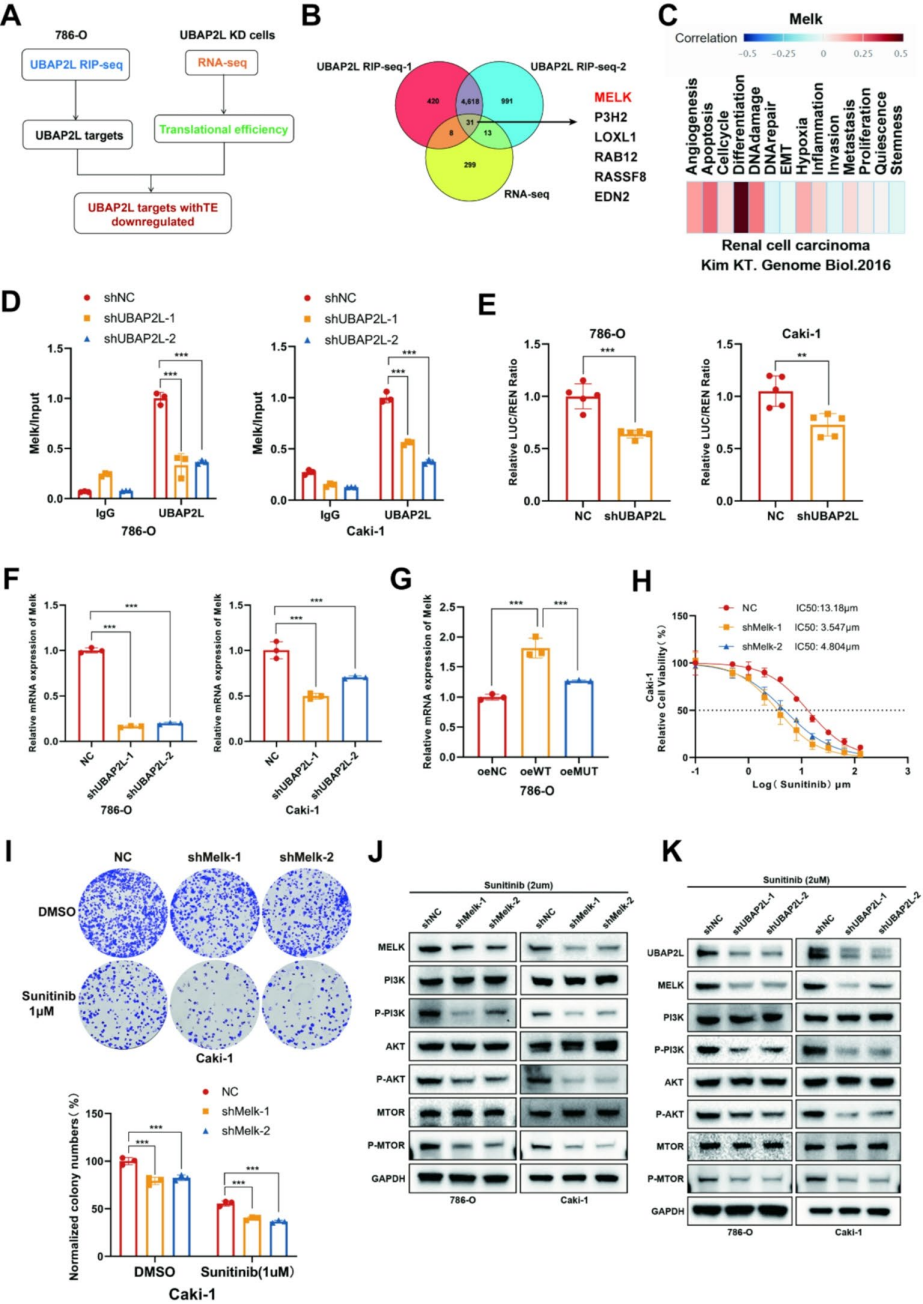
UBAP2L regulates mRNA stability of Melk activating the PI3K signaling pathways to enhance sunitinib resistance in ccRCC. (**A**) Schematic diagram of the strategy for pinpointing key UBAP2L targets in ccRCC. (**B**) Venn diagram illustrating the intersection among UBAP2L-bound genes by UBAP2L RIP seq and downregulated genes after UBAP2L knockdown (KD). (**C**) The cancerSEA dataset was used to analysis the cancer-related function of Melk. (**D**) Melk-specific qPCR analysis of the coprecipitated RNAs by UBAP2L antibodies in UBAP2L knockdown ccRCC cells. *P* values were determined by two-tailed t test or two-way ANOVA. ***P < 0.001. (**E**) Relative Fluc activity of wild-type Melk reporters in UBAP2L knockdown ccRCC cells. *P* values in results were determined by two-tailed unpaired t test, by one-way ANOVA. **P < 0.01, ***P < 0.001. (**F-G**) mRNA level of Melk in UBAP2L knockdown, overexpressed, or mutant-overexpressed ccRCC cells. *P* values were determined by two-tailed t test or two-way ANOVA. ***P < 0.001. (**H**) Caki-1 cells transfected with shMelk were treated with a serial dose of sunitinib for 24 h and subjected to CCK-8 assay. The IC50 values of sunitinib in each group were indicated. (**I**) Caki-1 cells infected with shMelk were harvested for colony formation assay after 2-week sunitinib treatment, Data presents as mean ± SEM with three replicates. Ns, not significant, ***P < 0.001. (**J**) The levels of PI3K pathway associated protein: PI3K, AKT, mTOR, p-PI3K, p-AKT and p-mTOR were detected in Melk down-regulated ccRCC cells treated with sunitinib (2µM). (**K**) The levels of Melk and PI3K pathway associated protein: PI3K, AKT, mTOR, p-PI3K, p-AKT and p-mTOR were detected in UBAP2L down-regulated ccRCC cells treated with sunitinib (2µM)

To decipher the mechanism through which the UBAP2L modulates sunitinib resistance in ccRCC, we conducted multiomics analysis to pinpoint downstream targets regulated by UBAP2L. UBAP2L-binding transcripts were determined by UBAP2L RNA immunoprecipitation sequencing (RIP-seq) analyses (Fig. 8A), and then were intersected with transcripts of decreased translational efficiency (TE) identified by RNA-sequencing (RNA-seq) in UBAP2L knockdown 786-O cells compared with controls cells (Fig. 8A-B). We identified 31 UBAP2L targets with TE downregulated (fold change < 0.5, *P* < 0.05) after UBAP2L knockdown. To further validate the impact of these 31 UBAP2L targets gene on sunitinib therapy, we knocked down four main genes: Melk, P3H2, LOXL1, and RAB12. By monitoring cell proliferation rates in the 786-O, 786-O-R, Caki-1, and Caki-1-R cell lines, we found that the knockdown of Melk reduced the sunitinib IC50 and reversed resistance, whereas P3H2, LOXL1, and RAB12 did not exhibit similar effects (Fig. S7A-F). Meanwhile, multiomics analysis (RNA-seq and RIP-seq) suggested that Melk could be a critical target of the UBAP2L axis. Subsequently, we employed the CancerSEA dataset (cancer single-cell state atlas) to investigate the function of Melk in various malignant tumors (Fig. 8C). Our analysis revealed a positive correlation between Melk expression and both hypoxia and angiogenesis, which may explain its association with resistance to tyrosine kinase inhibitors (TKIs), such as sunitinib, in ccRCC.

To determine whether there exists a bona fide interaction between UBAP2L and Melk, the RIP assay confirmed the direct binding of UBAP2L to the Melk transcript (Fig. 8D). Additionally, the dual-luciferase reporter gene assay further established that Melk might be a direct downstream target of UBAP2L (Fig. 8E). Then, to investigate the regulatory mechanism of Melk expression by UBAP2L, knockdown of UBAP2L in 786-O and Caki-1 cells resulted in decreased mRNA levels of Melk, while UBAP2L-WT overexpression, but not UBAP2L-MUT, increased the expression of Melk mRNA in the same cell lines (Fig. 8F-G and S8A).

The protein expression of Melk and its target genes, including p-PI3K, p-AKT, and p-mTOR, was significantly reduced following knockdown of either Melk or UBAP2L in ccRCC cell lines (Fig. 8J-K). Collectively, our in vitro and in vivo data indicate that UBAP2L enhances the expression of the key gene Melk by maintaining its mRNA stability and increasing its translational efficiency. CCK-8 assays, colony formation assays, and subcutaneous tumor formation experiments demonstrated that Melk knockdown markedly increased sensitivity to sunitinib in ccRCC cells both in vitro and in vivo (Fig. 8H-I and S8B-C).

**Fig. 9:**
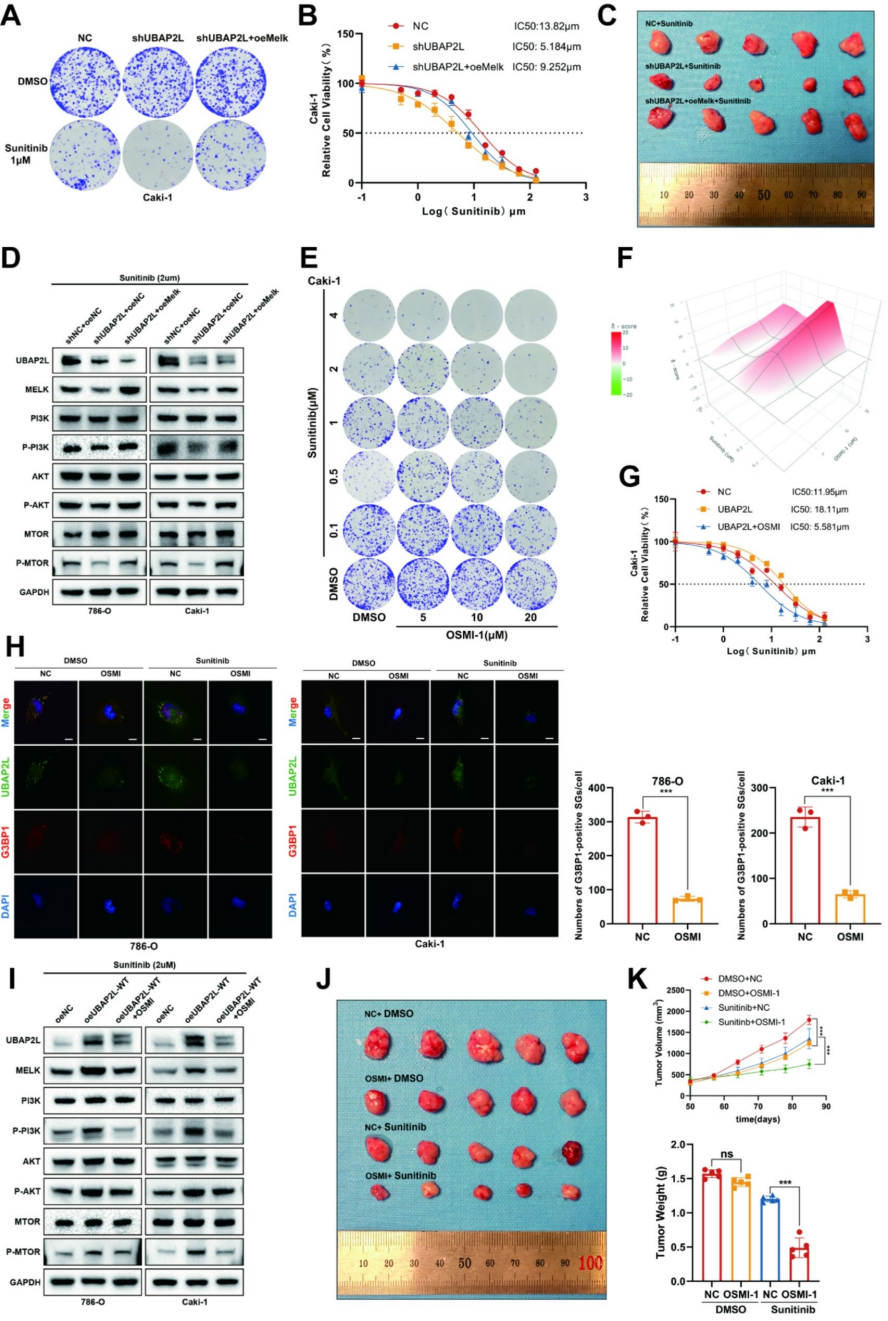
Ectopic expression of Melk rescues ccRCC sunitinib resistance in UBAP2L knockdown in vitro and in vivo and pharmacological decreases in the O-GlcNAcylation of UBAP2L inhibits stress granule formation and restores the sunitinib sensitivity of ccRCC. (**A**) Caki-1 control or UBAP2L knockdown cells with or without Melk overexpression were harvested for colony formation assay after 2-week sunitinib treatment. (**B**) Caki-1 control or UBAP2L knockdown cells with or without Melk overexpression were treated with a serial dose of sunitinib for 24 h and subjected to CCK-8 assay. The IC50 values of sunitinib in each group were indicated. (**C**) Representative images of xenograft ccRCC model for Melk rescue. 786-O control or UBAP2L knockdown cells with or without Melk overexpression were subcutaneously injected into the left flank of NOD mice (5 mice per group). (**D**) Western blot analysis of UBAP2L, Melk, Melk target genes PI3K, AKT, mTOR, p-PI3K, p-AKT and p-mTOR expression in Melk rescue assays in UBAP2L knockdown ccRCC cells. (**E**) Representative colony growth images of Caki-1 cells after combination treatment of OSMI-1 and sunitinib indicated. (**F**) 3D synergy maps of HSA scores between OSMI-1 and sunitinib are shown. (**G**) Caki-1 overexpression cells treated with or without 50µM OSMI-1 were treated with a serial dose of sunitinib for 24 h and subjected to CCK-8 assay. The IC50 values of sunitinib in each group were indicated. (**H**) 786-O and Caki-1 were treated with 2µM sunitinib and 50µM OSMI-1 for 24 h were immunostained for UBAP2L together with stress granules marker G3BP1, Scale bars, 10 μm. The number of G3BP1-positive stress granules per cell was calculated using ImageJ software. Data presents as mean ± SEM with three replicates. ***P < 0.001. (**I**) Western blot analysis of UBAP2L, Melk, Melk target genes PI3K, AKT, mTOR, p-PI3K, p-AKT and p-mTOR expression in OSMI-1 rescue assays in UBAP2L overexpression ccRCC cells. (**J**) Representative images of xenograft ccRCC model (5 mice per group). (**K**) Tumor weight measured after surgical dissection. Tumor volume measured weekly during tumor growth. Data are shown as mean ± SEM. Ns, not significant, ***P < 0.001

To validate whether Melk acts as a downstream effector of UBAP2L in regulating ccRCC sunitinib resistance, we performed rescue assays by overexpressing Melk in UBAP2L knockdown 786-O and Caki-1 cells. Functional assays revealed that Melk overexpression significantly promoted proliferation and drug resistance to sunitinib in UBAP2L knockdown ccRCC cells (Fig. 9A-C and S8D-H). Furthermore, Melk restored the protein expression of PI3K pathway genes in UBAP2L knockdown ccRCC cells (Fig. 9D). Collectively, these data further support the notion that UBAP2L deficiency disrupts ccRCC sunitinib resistance by compromising Melk expression.

### Pharmacological decreases in the O-GlcNAcylation of UBAP2L restores the sunitinib sensitivity of ccRCC

To translate our findings into a clinical context, we investigated the effects of reduced O-GlcNAcylation of UBAP2L in ccRCC. Our results indicated that OSMI-1, an inhibitor of OGT, significantly decreased O-GlcNAcylation in ccRCC cells, as well as the expression levels of UBAP2L and components of the Melk-PI3K-AKT-mTOR signaling pathway (Fig. 9I). Colony formation assays and CCK-8 assays demonstrated that the combination of sunitinib and OSMI-1 effectively inhibited cell growth in ccRCC cells (Fig. 9E-G and S8J-K).

Furthermore, we examined the impact of OSMI-1 on stress granule (SG) formation. 786-O and Caki-1 cells were treated with OSMI-1 and sunitinib, followed by immunostaining for UBAP2L and G3BP1. As illustrated in Fig. 9H and S9A-B, OSMI-1 markedly disrupted SG organization, corroborating previous findings.

In line with our in vitro results, the patient-derived xenograft (PDX) ccRCC mouse model receiving 1 mg/kg OSMI-1 treatment for 40 days (administered every two days from day 30 to day 70 post-tumor implantation) exhibited slower tumor growth and enhanced survival compared to the vehicle-treated control group (Fig. 9J-K).

Additionally, we utilized a patient-derived organoid (PDO) model to further investigate the function of OSMI-1. As illustrated in Fig. S8L, the combination treatment of sunitinib and OSMI-1 significantly inhibited the growth of PDOs, thereby underscoring the critical role of OSMI-1 in enhancing sunitinib sensitivity in ccRCC.

Collectively, these findings indicate that OSMI-1 treatment may effectively suppress stress granule formation by reducing UBAP2L O-GlcNAcylation. This suppression subsequently decreases the mRNA stability of Melk and inhibits the PI3K signaling pathways, thereby increasing sunitinib sensitivity in clear cell renal cell carcinoma.

## Discussion

Sunitinib has served as a cornerstone in the treatment of metastatic ccRCC for over 10 years. However, the molecular mechanisms underlying both intrinsic and acquired resistance remain poorly understood. In our study, we developed sunitinib-resistant PDX models and identified UBAP2L as a critical mediator of sunitinib resistance. This research uncovers a novel regulatory axis that O-GlcNAcylation of UBAP2L promoted its protein stability via inhibiting TRIM37-mediated ubiquitination and regulated stress granule formation, thereby enhancing the mRNA stability of Melk and activating the PI3K signaling pathway.

Previous research has implicated UBAP2L in drug resistance across various cancer types. However, its role in ccRCC has not been previously defined. In this study, we demonstrate that UBAP2L is upregulated in sunitinib-resistant ccRCC PDX models, clinical tissues, and cell lines. Functional validation indicates that its overexpression promotes sunitinib resistance both in vitro and in vivo. This finding is consistent with the established roles of UBAP2L in enhancing tumor progression through pathways such as PI3K/AKT and NF-κB in gastric cancer, suggesting that its functions in oncogenesis are conserved yet context-specific [33].

A central finding of our work is the involvement of UBAP2L in orchestrating stress granule (SG) formation, a critical adaptive response that enables cancer cells to survive therapeutic stress. We show that UBAP2L acts as a core component of SGs in ccRCC, driving SG nucleation, thereby mediating sunitinib resistance. SGs function as hubs for mRNA storage and translational repression, fostering resistance by modulating apoptosis, stress tolerance, and interactions within the tumor microenvironment [34–36]. Notably, SGs sequester pro-apoptotic mRNAs, such as BAX and CASP3, inhibiting their translation while stabilizing the expression of anti-apoptotic proteins, including BCL2, to ensure the survival of tumor cells [37, 38]. These findings collectively underscore UBAP2L as a multifunctional regulator of SG biogenesis—an increasingly implicated mechanism of chemoresistance in various cancers, including hepatocellular and breast malignancies [27, 39].

O-GlcNAcylation has emerged as a critical regulator of UBAP2L stability. We identified OGT as the enzyme that mediates O-GlcNAcylation of UBAP2L at serine 305 (S305), a modification that protects UBAP2L from TRIM37-dependent ubiquitin-proteasomal degradation. The finding bridges two vital post-translational regulatory systems—O-GlcNAcylation and ubiquitination—in controlling UBAP2L levels, a balance that is pivotal for SG assembly and subsequent sunitinib resistance. Mechanistically, stabilized UBAP2L promotes resistance by enhancing Melk mRNA stability, thus activating the PI3K-AKT-mTOR pathway, a known driver of therapeutic resistance. O-GlcNAcylation enhances protein stability by inhibiting ubiquitination-mediated degradation, corroborating earlier findings. Specifically, O-GlcNAcylation interrupts the ubiquitination process and subsequent proteasomal degradation by obstructing the interaction between β-catenin and β-TrCP, a subunit of the E3 ubiquitin ligase complex, which ultimately leads to increased protein stability [40]. Furthermore, O-GlcNAcylation modifications at key sites, such as T236 of YY1 and S112 of SNAI1, directly compete with phosphorylation, inhibiting signaling from kinases like GSK3β and preventing degradation via ubiquitination [41, 42]. Collectively, these findings suggest that O-GlcNAcylation represents a potential therapeutic target for overcoming sunitinib resistance in ccRCC.

Clinically, targeting the OGT-UBAP2L axis with the small-molecule inhibitor OMSI-1 replicates reduced UBAP2L expression, suppressed PI3K-AKT-mTOR signaling, and restored sunitinib sensitivity in resistant ccRCC cells. This suggests that disrupting O-GlcNAcylation-dependent UBAP2L stabilization may represent a viable strategy to overcome sunitinib resistance. Our work aligns with emerging efforts to target SGs and post-translational modifications in cancer therapy, such as MPN-based SG inhibitors in hepatocellular carcinoma and G3BP2-targeted approaches in breast cancer, underscoring the translational potential of targeting stress response pathways [34, 43].

A limitation of this study is the need for a larger number of PDX and PDO models to validate our conclusions concerning the clinical application of drug-resistant targets. Furthermore, the role of stress granules in promoting tumor drug resistance may involve a dual mechanism, necessitating further investigation into the potential interactions between UBAP2L and stress granules. Additionally, O-GlcNAcylation is a dynamic and reversible modification, therefore, a more thorough exploration of the balance between O-GlcNAcylation and de-O-GlcNAcylation of UBAP2L in ccRCC is warranted.

## Conclusions

This study delineates a novel mechanism by which OGT-mediated O-GlcNAcylation of UBAP2L enhances stress granule formation and Melk mRNA stability, thereby driving sunitinib resistance in ccRCC. These findings not only advance our understanding of treatment resistance in renal cell carcinoma but also underscore UBAP2L and its regulatory modifications as promising targets for overcoming therapeutic challenges. Future research should investigate the interplay between UBAP2L-dependent SG functions and broader metabolic or immune pathways in ccRCC, as well as validate OGT inhibition in preclinical and clinical settings.

## Supplementary Information

Below is the link to the electronic supplementary material.

Supplementary Material 1

## Data Availability

No datasets were generated or analysed during the current study.
