# Supplementary material for: O-GlcNAcylation of UBAP2L regulates stress granule formation and sunitinib resistance in clear cell renal cell carcinoma
Source: J Exp Clin Cancer Res. 2025 Sep 30;44:273. doi: 10.1186/s13046-025-03534-0 (PMC12487280; doi:10.1186/s13046-025-03534-0)
Supplement: Supplementary file 1 — Supplementary Material 1 [file 13046_2025_3534_MOESM1_ESM.docx]

**Supplementary Materials for**

O-GlcNAcylation of UBAP2L regulates stress granule formation and sunitinib resistance in clear cell renal cell carcinoma

**Authors**

Jiajun Xing^1,a^, Baochao Li^1,a^, Songbo Wang^1,a^, Zengjun Wang^1,*^ Chenkui Miao^1,*^

**Affiliations**

1 Department of Urology, The First Affiliated Hospital of Nanjing Medical University, No 300 Guangzhou Road, Nanjing, China

***Corresponding Author Email:** [zengjunwang@njmu.edu.cn](mailto:zengjunwang@njmu.edu.cn), medicalmck@163.com

aThese authors contributed equally to this work.

**Competing interests**

Authors declare that they have no competing interests.

**This PDF file includes:** Figs. S1 to S9**,** Tables S1 to S2

**Supplementary Figures**

**FigureS1**

(A). Tumor volume measured weekly during ccRCC PDX tumor growth. Data are shown as mean ± SEM. Ns, not significant, ***P < 0.001.

(B). qPCR analysis of UBAP2L expression in sunitinib-resistant and sunitinib-sensitive ccRCC PDX models. Ns, not significant

(C). qPCR analysis of UBAP2L expression in sunitinib-resistant and sunitinib-sensitive ccRCC cell models. Ns, not significant, *P < 0.05.

(D). The relative protein expression level of UBAP2L from ccRCC PDX models with (n = 3) or without (n = 3) sunitinib resistance was calculated using ImageJ software.


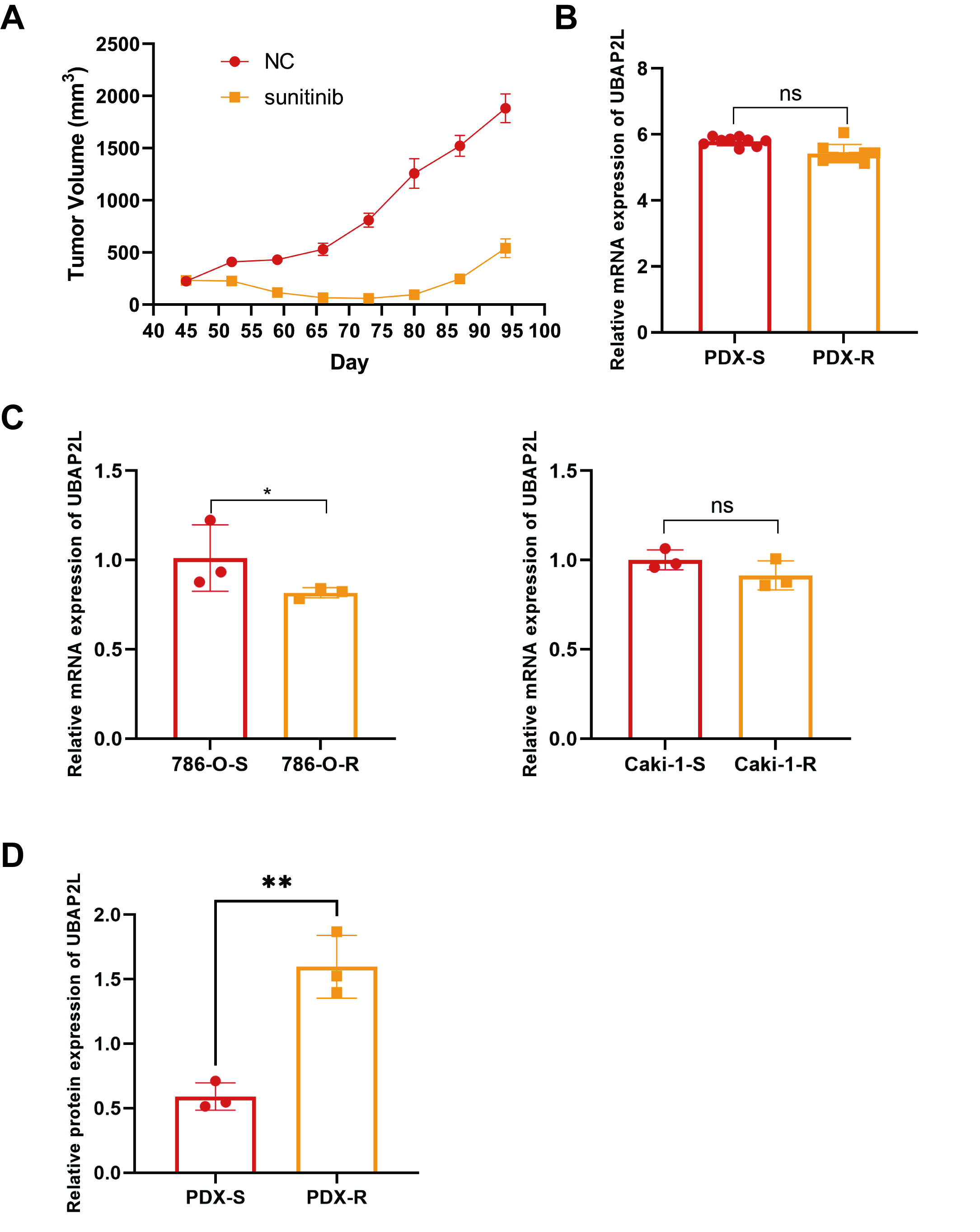


**FigureS2**

(A). 786-O-R and Caki-1-R cells were transfected with indicated constructs for 72h. After puromycin selection, these cells were treated with a serial dose of sunitinib for 24h, and subjected to CCK-8 assay. The IC50 values of sunitinib in each group were indicated.

(B). 786-O and Caki-1 cells were transfected with indicated constructs for 72h. After puromycin selection, these cells were treated with a serial dose of sunitinib for 24h, and subjected to CCK-8 assay. The IC50 values of sunitinib in each group were indicated.


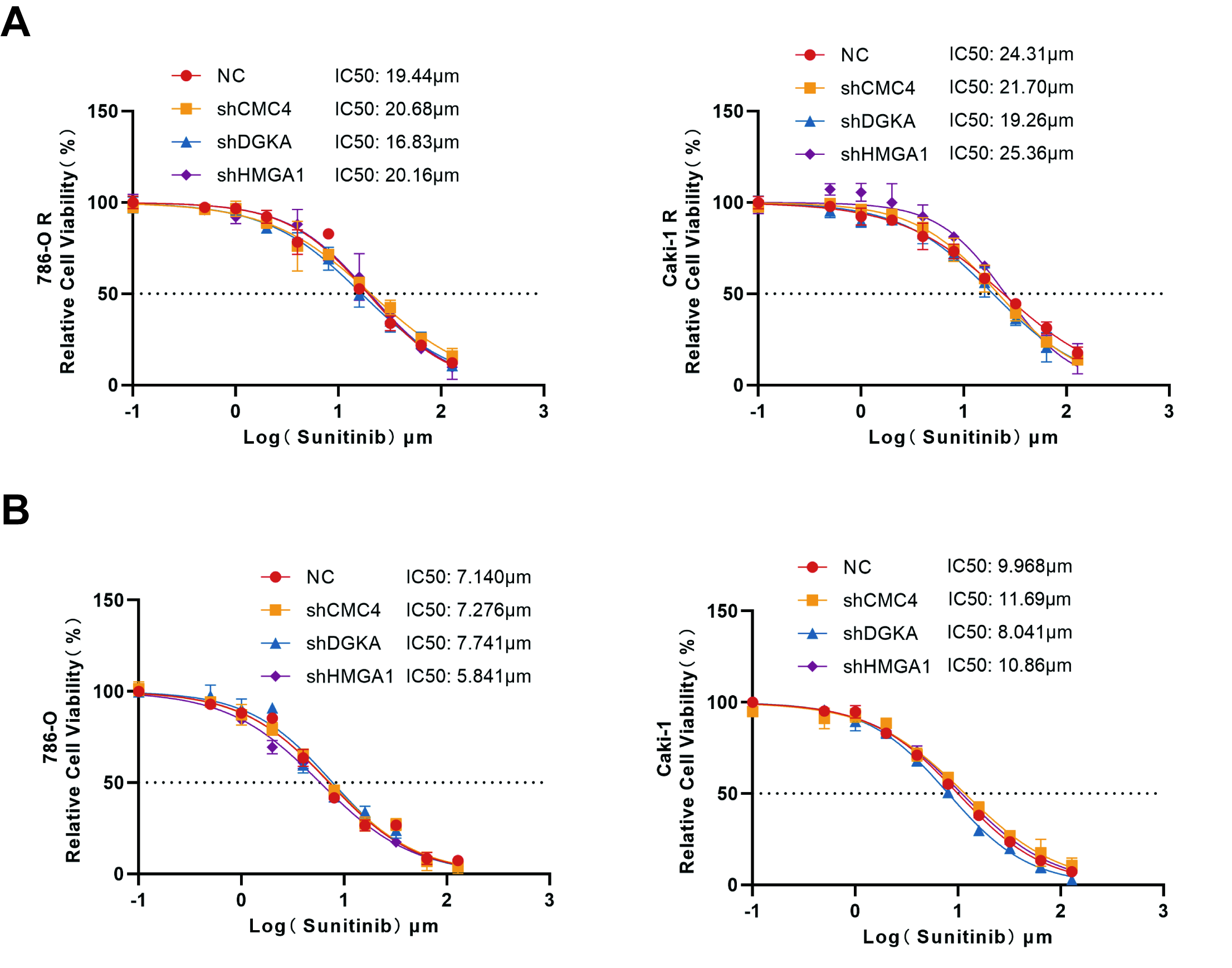


**FigureS3**

(A). 786-O-R and Caki-1-R cells were transfected with indicated constructs for 72h. After puromycin selection, cells were collected for Western blot analysis.

(B). 786-O and Caki-1 cells were transfected with indicated constructs for 72h. After puromycin selection, cells were collected for Western blot analysis.

(C-E). Caki-1 and Caki-1-R cells were transfected with indicated constructs for 72h. After puromycin selection, these cells were treated with a serial dose of sunitinib for 24h. and subjected to CCK-8 assay. The IC50 values of sunitinib in each group were indicated.

(F). Caki-1 control or UBAP2L knockdown cells were treated with or without sunitinib (2μM) for 96h and subjected to CCK-8 assay. P values were determined by two-tailed t test or two-way ANOVA. ***P < 0.001.

(G). Colony formation assays upon UBAP2L knockdown Caki-1 cells after 2-week sunitinib treatment. Ns, not significant, ***P < 0.001.

(H). Colony formation assays upon UBAP2L knockdown 786-O-R cells after 2-week sunitinib treatment. Ns, not significant, ***P < 0.001.

(I). Colony formation assays upon UBAP2L knockdown Caki-1-R cells after 2-week sunitinib treatment. Ns, not significant, ***P < 0.001.


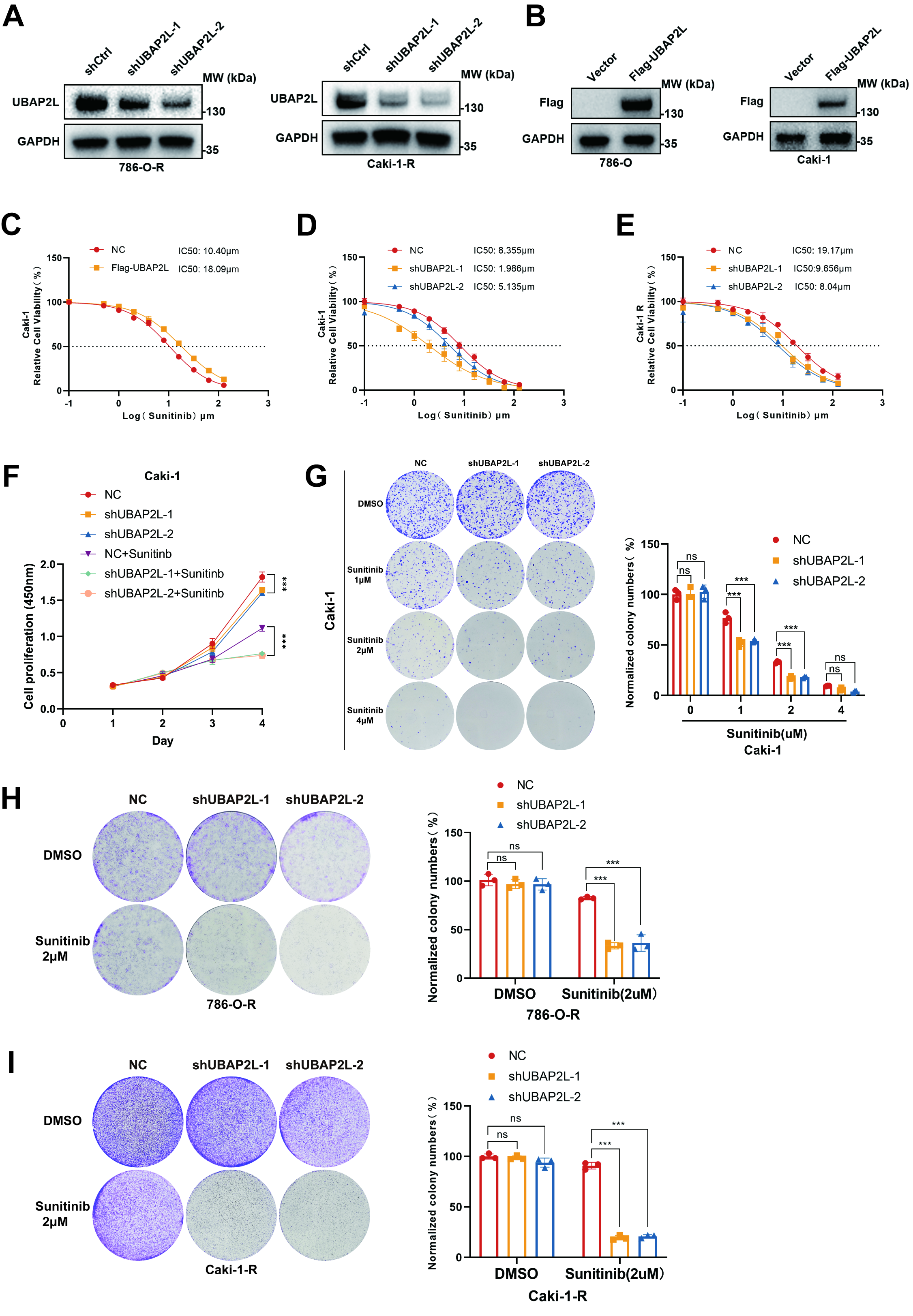


**FigureS4**

(A-F). Caki-1 cells were transfected with indicated constructs for 72 h. After 24h puromycin selection, cells were treated with or without sunitinib (2μM or 4μM) for another 24h. Cells were collected for Caspase 3 activity assay (A), Flow cytometry analysis of apoptosis (B), Western blot analysis (C-D) and TUNEL staining (F). Scale bars, 150μm. The number of Tunel-positive cell was calculated using ImageJ software (E-F).

(G). The number of G3BP1-positive stress granules per cell was calculated using ImageJ software. P values were determined by two-tailed t test or two-way ANOVA. ***P < 0.001.

(H). Caki-1 was transfected with control or UBAP2L shRNAs, and 72 h later, and the cells treated with 2μM sunitinib for 24h were immunostained for UBAP2L together with G3BP1, Scale bars, 10μm.

(I). The accumulation of G3BP1 after UBAP2L knockdown and sunitinib treatment in Caki-1. SG fractions (S850) were obtained by serial centrifugations, and the samples were analyzed by immunoblotting with the indicated antibodies. Protein levels of G3BP1 and UBAP2Lwere quantified. GAPDH was used as the loading control.


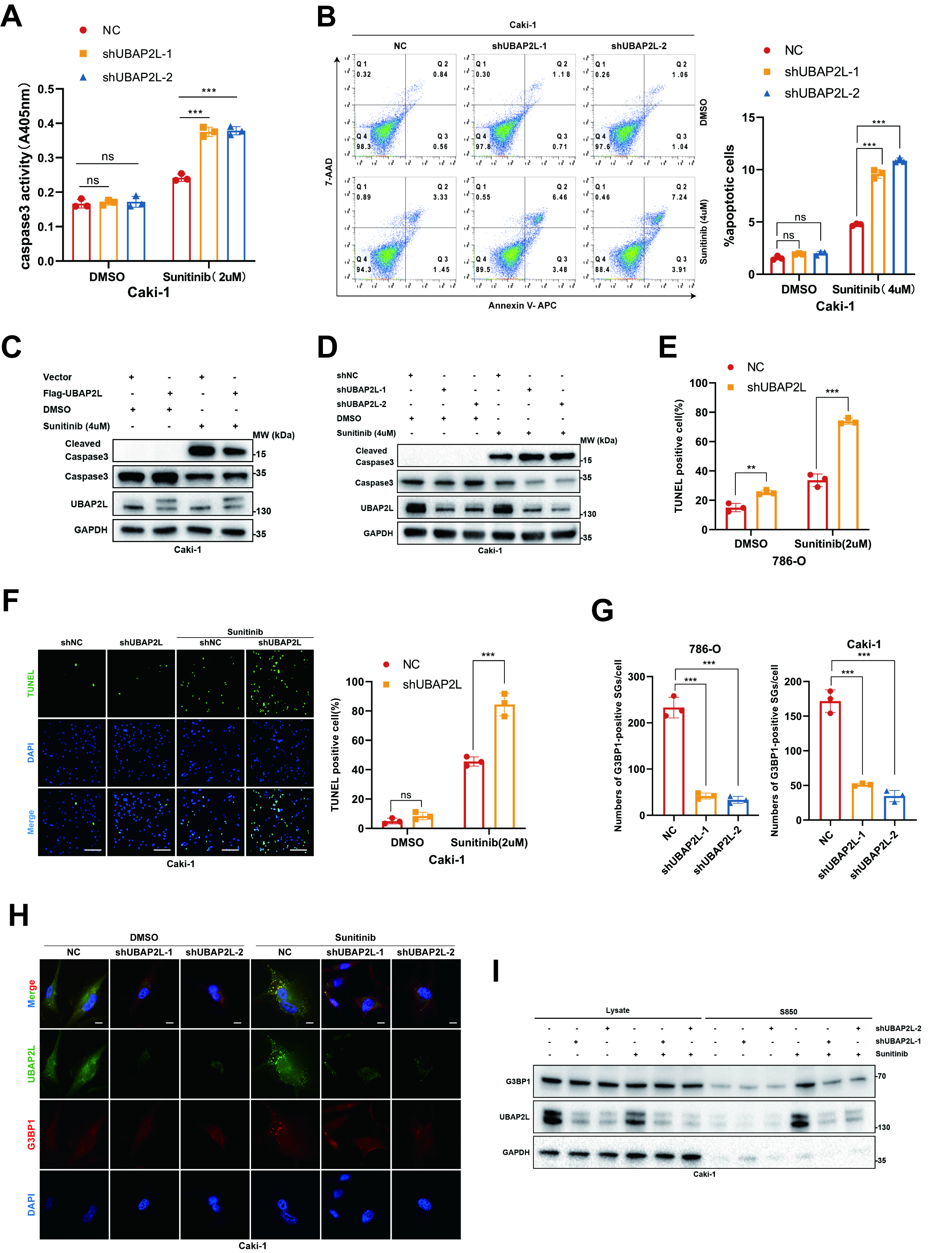


**FigureS5**

(A). Mass spectrometry analysis of a peptide derived from Flag-UBAP2L-immunoprecipitates to show the potential interaction between UBAP2L and OGT.

(B-C). Red (UBAP2L) and green (OGT) curves depict the fluorescence intensity of two distinct signals as a function of distance (0-50μm) in 786-O and Caki-1 cells.

(D). Caki-1 cells infected with Flag-UBAP2L WT/S305A were treated with or without sunitinib (2μM) for 96h and subjected to CCK-8 assay. P values were determined by two-tailed t test or two-way ANOVA. ***P < 0.001.

(E). Caki-1 cells infected with Flag-UBAP2L WT/S305A were harvested for colony formation assay after 2-week sunitinib treatment, P values were determined by two-tailed t test or two-way ANOVA. Ns, not significant, ***P < 0.001.

(F). The number of branches points in each group from Figure 5E were quantified using the Image J software. Data presents as mean ± SEM with three replicates. ***P < 0.001.

(G-H). Tumor weight (H) measured after surgical dissection. Tumor volume (G) measured weekly during tumor growth. Data are shown as mean ± SEM. *P < 0.05, P < 0.01 ***P < 0.001.


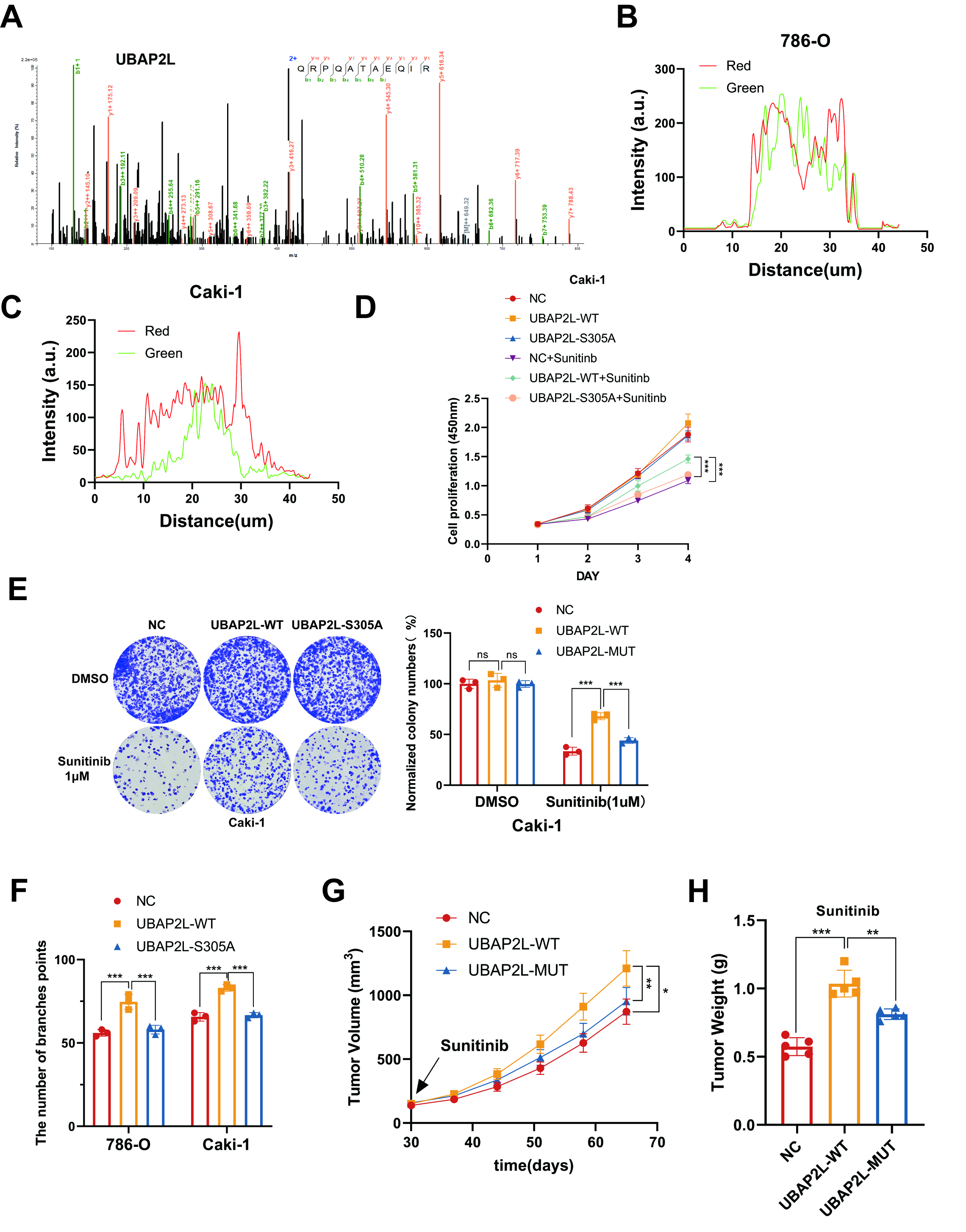


**FigureS6**

(A-B). The accumulation of G3BP1 after sunitinib treatment in Caki-1 or 786-O cells infected with Flag-UBAP2L WT/S305A. SG fractions (S850) were obtained by serial centrifugations, and the samples were analyzed by immunoblotting with the indicated antibodies. Protein levels of G3BP1 and UBAP2Lwere quantified. GAPDH was used as the loading control.

(C). Caki-1 cells were treated with or without the proteasome inhibitor MG132 (10 μM,0-12h) and the autophagy inhibitor 3-MA (10μM, 0-12h), and then UBAP2L were detected.

(D). Lysates from ccRCC cells were subjected to IP and immunoblotting analysis with the indicated antibodies.

(E). Immunofluorescence confocal microscopy showed the colocalization of UBAP2L and TRIM37 in 786-O and Caki-1 cells. Scale bar, 10μm.

(F). Red (UBAP2L) and green (TRIM37) curves depict the fluorescence intensity of two distinct signals as a function of distance (0-50μm) in 786-O and Caki-1 cells.

(G). qPCR analysis of OGT and OGA mRNA expression in sunitinib-resistant and sunitinib-sensitive ccRCC cell models. Ns, not significant, *P < 0.05.

(H). Caki-1 cells transfected with the shCtrl, shTRIM37, TRIM37-WT or TRIM37-MUT were treated with CHX (50µg/ml), and collected at the indicated times for Western Blot.


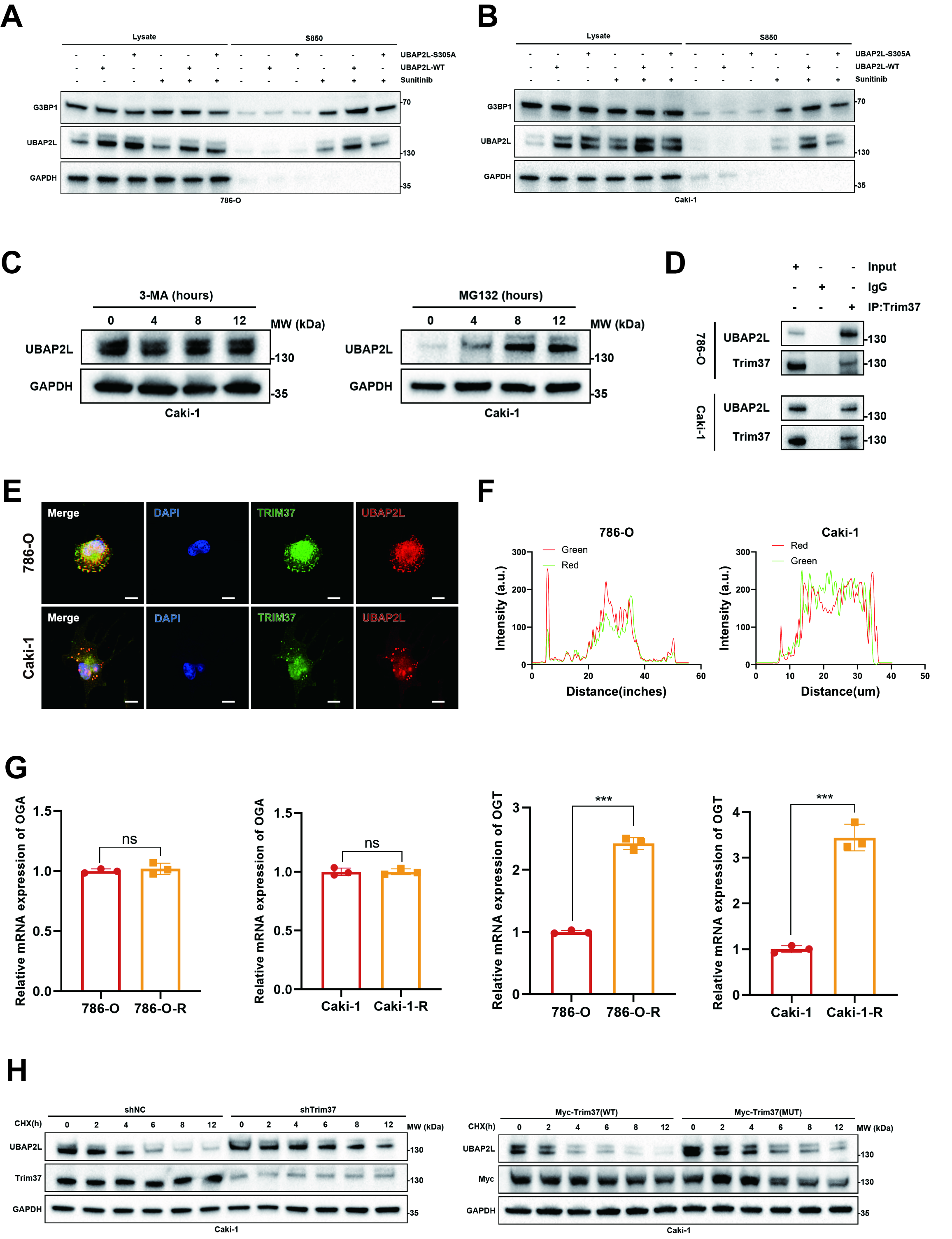


**FigureS7**

(A-B). 786-O-R and Caki-1-R cells were transfected with indicated constructs for 72h. After puromycin selection, these cells were treated with a serial dose of sunitinib for 24h. and subjected to CCK-8 assay. The IC50 values of sunitinib in each group were indicated.

(C-D). 786-O and Caki-1 cells were transfected with indicated constructs for 72h. After puromycin selection, these cells were treated with a serial dose of sunitinib for 24h. and subjected to CCK-8 assay. The IC50 values of sunitinib in each group were indicated.

(E-F). Melk knockdown 786-O-R and Caki-1-R cells were treated with a serial dose of sunitinib for 24h. and subjected to CCK-8 assay. The IC50 values of sunitinib in each group were indicated.

**
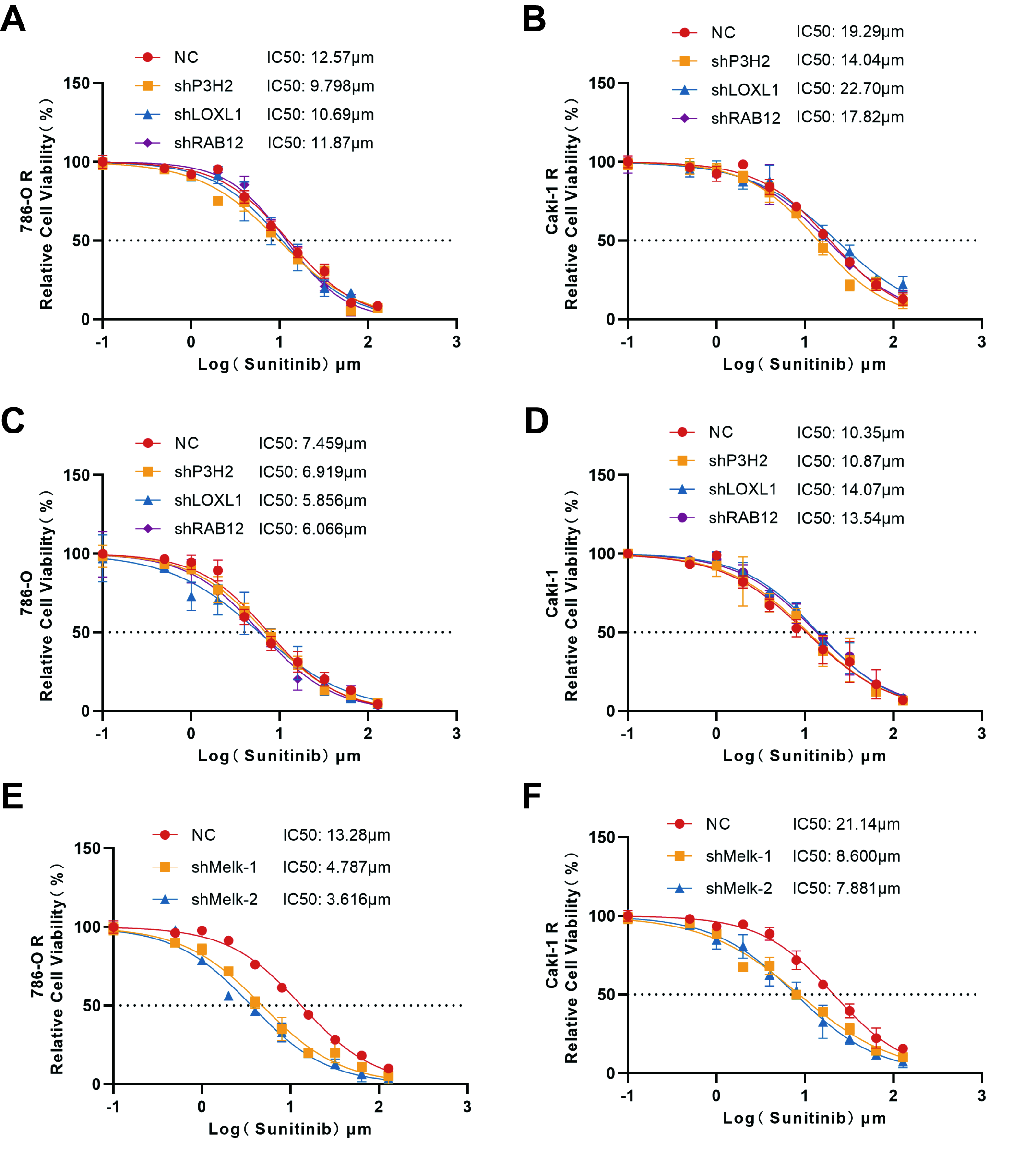
**

**FigureS8**

(A). mRNA level of Melk in UBAP2L overexpressed and mutant-overexpressed Caki-1 cells. P values were determined by two-tailed t test or two-way ANOVA. ***P < 0.001.

(B). 786-O cells infected with shMelk were harvested for colony formation assay after 2-week sunitinib treatment, Ns, not significant, ***P < 0.001.

(C). 786-O cells transfected with shMelk were treated with a serial dose of sunitinib for 24h and subjected to CCK-8 assay. The IC50 values of sunitinib in each group were indicated.

(D). 786-O control or UBAP2L knockdown cells with or without Melk overexpression were harvested for colony formation assay after 2-week sunitinib treatment.

(E). The normalized colony numbers were was calculated using ImageJ software. P values were determined by two-tailed t test or two-way ANOVA. ***P < 0.001.

(F). 786-O control or UBAP2L knockdown cells with or without Melk overexpression were treated with a serial dose of sunitinib for 24h and subjected to CCK-8 assay. The IC50 values of sunitinib in each group were indicated.

(G-H). Tumor weight measured after surgical dissection. Tumor volume measured weekly during tumor growth. Data are shown as mean ± SEM. Ns, not significant, ***P < 0.001.

(I). The levels of Melk and PI3K pathway associated protein: PI3K, AKT, mTOR, p-PI3K, p-AKT and p-mTOR were detected in UBAP2L overexpressed, or mutant-overexpressed ccRCC cells treated with sunitinib (2μM).

(J). Representative colony growth images of 786-O cells after combination treatment of OSMI-1 and sunitinib indicated. 3D synergy maps of HSA scores between OSMI-1 and sunitinib are shown.

(K). 786-O overexpression cells treated with or without 50μM OSMI-1 were treated with a serial dose of sunitinib for 24h and subjected to CCK-8 assay. The IC50 values of sunitinib in each group were indicated.

(L). Representative images of ccRCC PDOs after 50μM OSMI-1 and 2μM sunitinib treatments for 4 days, the morphology and size of the organoids were observed and analyzed. Scale bar, 50µm


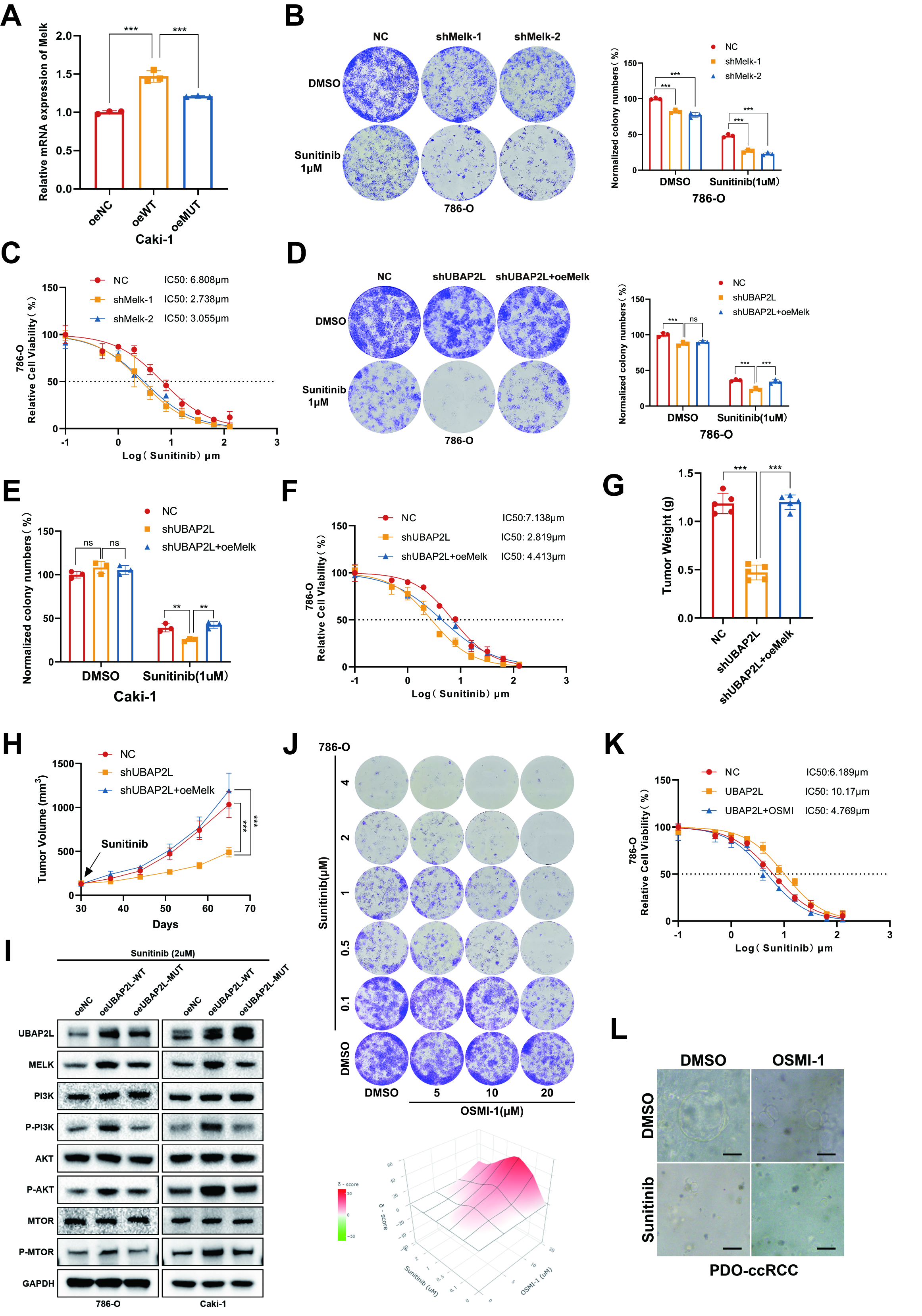


**FigureS9**

(A-B). The accumulation of G3BP1 after sunitinib treatment in Caki-1 and 786-O cells with or without OSMI-1. SG fractions (S850) were obtained by serial centrifugations, and the samples were analyzed by immunoblotting with the indicated antibodies. Protein levels of G3BP1 and UBAP2Lwere quantified. GAPDH was used as the loading control.


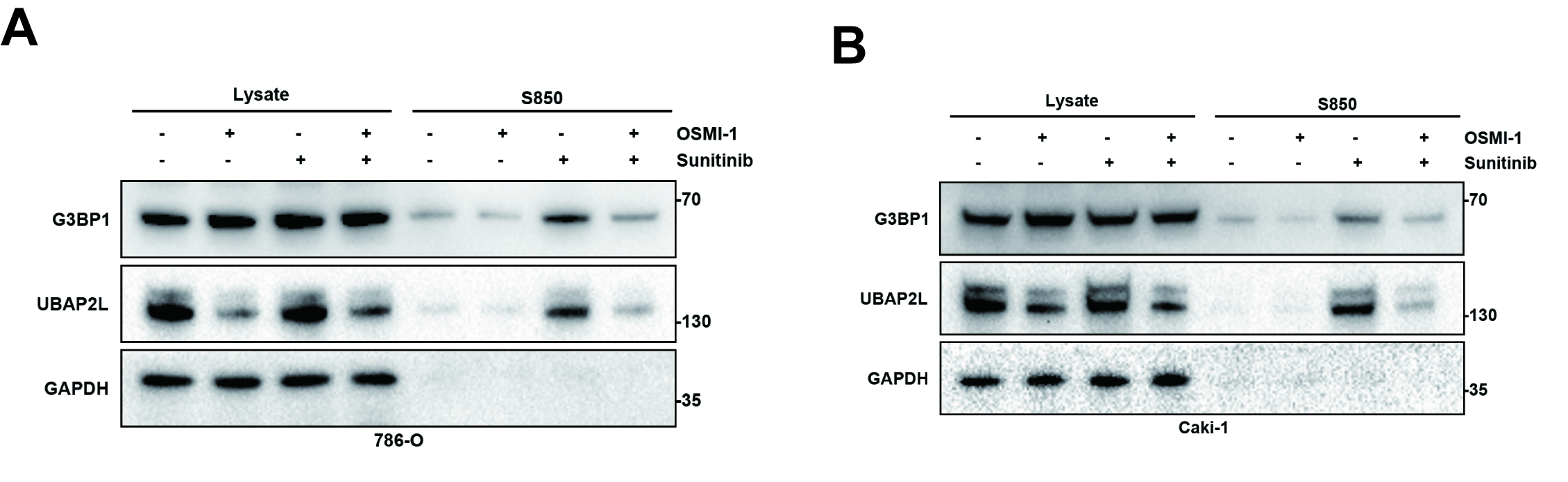


**Supplementary Tables**

**TableS1**

| shRNA and sgRNA | 5′- sequences-3′ |
| --- | --- |
| si-UBAP2L-1 | GGAGAUGUCAACAGAGCUAUCTT |
| si-UBAP2L-2 | AGUUCUUGGAGCAAUUCAAGATT |
| si-OGT-1 | GCCUGAUAGAUCUGGCAAUTT |
| si-OGT-2 | CGCGUGCCAUCCAAAUUAATT |
| si-OGA-1 | GGGACAUCAAGAGUAUAAUTT |
| si-OGA-2 | GCCAAUUGAUGGAGCAAAUTT |
| shTRIM37-1 | GCTGAAGAATAAGCTTATA |
| shTRIM37-2 | GCTACGAGAACTAGTAAAT |
| sgTRIM37 | GTCCTGAAACTTGAAGAGG |
| shP3H2 | CCCAAGATAGATCGAGACCTA |
| shLOXL1 | CATTCACTACACAGGTCGCTA |
| shRAB12 | CTGTACCTTTGTAATGATAAA |
| shCMC4 | CCATGTTTCCACCAAATGAAT |
| shDGKA | CGGCCAGAAGACAAGTTAGAA |
| shHMGA1 | CAACTCCAGGAAGGAAACCAA |

**TablesS2**

| primers | sequences |
| --- | --- |
| UBAP2L-F | ACGCAGCAGACATTCCTGAA |
| UBAP2L-R | GAACACAGCAGGCCCATACT |
| Melk-F | TCTCCCAGTAGCATTCTGCTT |
| Melk-R | AGAAAGCCTTAAACGAACTGGTT |
| GAPDH-F | AATGGGCAGCCGTTAGGAAA |
| GAPDH-R | GCCCAATACGACCAAATCAGAG |
| OGA-F | GAAGGAGAGTCAAGCGACGTT |
| OGA-R | TCCATAACCCAAGGTCTTCCAT |
| P3H2-F | TTGAAGCATTGCAGTTGGTAGA |
| P3H2-R | GCCTGATAGCCATCTCAAAGTC |
| LOXL1-F | CTGTGCTGCGGAGGAGAAG |
| LOXL1-R | GTAGTGGCTGAACTCGTCCA |
| RAB12-F | CTGGGATGCGGTTCTGTGAAG |
| RAB12-R | CAGTTCTGGCGGTATCTCAGG |
| CMC4-F | TCCAAGAACTGCGTAAGTGTTG |
| CMC4-R | GCAGACTTCCGTGTTAGGTTTTC |
| DGKA-F | AGGATGGCGAGATGGCTAAAT |
| DGKA-R | CCAGGCTTAGGTGTCTGGG |
| HMGA1-F | GCTGGTAGGGAGTCAGAAGGA |
| HMGA1-R | TGGTGGTTTTCCGGGTCTTG |
| OGT-F | TTCCGAGTGAAGGTGATGGC |
| OGT-R | CCCAGCCACATTGCCTGAAT |
